# A New Logic, a New Information Measure, and a New Information-Based Approach to Interpreting Quantum Mechanics

**DOI:** 10.3390/e26020169

**Published:** 2024-02-15

**Authors:** David Ellerman

**Affiliations:** Faculty of Social Sciences, University of Ljubljana, 1000 Ljubljana, Slovenia; david@ellerman.org

**Keywords:** logic of partitions, logical entropy, ontic indefiniteness, indistinguishability

## Abstract

The new logic of partitions is dual to the usual Boolean logic of subsets (usually presented only in the special case of the logic of propositions) in the sense that partitions and subsets are category-theoretic duals. The new information measure of logical entropy is the normalized quantitative version of partitions. The new approach to interpreting quantum mechanics (QM) is showing that the mathematics (not the physics) of QM is the linearized Hilbert space version of the mathematics of partitions. Or, putting it the other way around, the math of partitions is a skeletal version of the math of QM. The key concepts throughout this progression from logic, to logical information, to quantum theory are distinctions versus indistinctions, definiteness versus indefiniteness, or distinguishability versus indistinguishability. The distinctions of a partition are the ordered pairs of elements from the underlying set that are in different blocks of the partition and logical entropy is defined (initially) as the normalized number of distinctions. The cognate notions of definiteness and distinguishability run throughout the math of QM, e.g., in the key non-classical notion of superposition (=ontic indefiniteness) and in the Feynman rules for adding amplitudes (indistinguishable alternatives) versus adding probabilities (distinguishable alternatives).

## 1. Introduction: Where Does QM Math Come from?

The mathematics of quantum mechanics (QM) is quite distinctive and different from the mathematics of classical physics. Where does that distinctive math of QM come from? The purpose of this paper is to expound a new way to approach that distinctive mathematics. The result is a new corroboration of an existing realistic interpretation of QM. The new approach to understanding the math of QM starts with a new form of mathematical logic, the logic of partitions or equivalence relations [1], which is dual to the usual Boolean logic of subsets. The quantitative form of that logic gives the notion of logical entropy that provides a logical notion of information-as-distinctions (or information-as-distinguishability) [2].

This approach can be situated in the program of reconstructing QM with “information” playing a key role, as surveyed by Gregg Jaeger [3]. The approaches surveyed by Jaeger, with one partial exception, either use “information” in a non-technical meaning or refer to Shannon’s notion of entropy. The exception is Bruckner and Zeilinger [4] who suggest the correct formula for logical entropy without the derivation as the normalized quantitative measure of how much a partition distinguishes—or in complementary terms, how much an equivalence relation ‘indistinguishes’. Shannon explicitly said that his entropy was not a definition of information, i.e., “no concept of information itself was defined” in communications theory [5] [p. 458], but at best it is a measure or quantification of information.

In our approach, only the math of QM is reconstructed as the Hilbert space version of the math of partitions—and that alone is sufficient to see the type of objectively indefinite ontology described by that math. Quantization, then, shows the changes in classical physics to fit that new math framework, e.g., the De Broglie and Einstein relations. This “cutting-at-the-joints” separation between the math and physics is evidenced by Planck’s constant having no role in the math argument correlating partition math with QM math.

In some other approaches to the philosophy of QM, the modus operandi seems to be that the standard theory needs to be changed in order to be interpreted, e.g., Bohmian mechanics or spontaneous localization. The approach taken here is to further corroborate a realistic interpretation of the standard non-relativistic finite-dimensional von Neumann/Dirac theory.

## 2. Materials and Methods

The key mathematical, indeed logical, concept is the notion of a partition on a set—or equivalently, the notion of an equivalence relation. The basic thesis is that *the math of QM is the Hilbert space version of the math of partitions*. Partitions are the basic logical concept to describe the following:

- Distinctions versus indistinctions,
- Definiteness versus indefiniteness,
- Distinguishability versus indistinguishability

These are the concepts that define information-as-distinctions (or distinguishability). That is, the new logical information measure, logical entropy, is just the normalized quantitative version of partitions.

The key non-classical notion of state in QM is that of *superposition*—with entanglement being a particularly unintuitive special case.

Thus, *superposition*, with the attendant riddles of entanglement and reduction, remains *the* central and generic interpretative problem of quantum theory [6] [p. 27]

A simplified slogan might be: “Superposition = equivalence” in the sense that equivalence at the set level prefigures superposition at the quantum level. The result of the thesis is to give a partitional or equivalence-relation explication of superposition in terms of objective indefiniteness between definite alternatives—so that this approach to QM could be called the *Objective Indefiniteness Interpretation* of QM.

From these two basic ideas alone—indefiniteness and the superposition principle—it should be clear already that quantum mechanics conflicts sharply with common sense. If the quantum state of a system is a complete description of the system, then a quantity that has an indefinite value in that quantum state is objectively indefinite; its value is not merely unknown by the scientist who seeks to describe the system. Furthermore, since the outcome of a measurement of an objectively indefinite quantity is not determined by the quantum state, and yet the quantum state is the complete bearer of information about the system, the outcome is strictly a matter of objective chance—not just a matter of chance in the sense of unpredictability by the scientist. Finally, the probability of each possible outcome of the measurement is an objective probability. Classical physics did not conflict with common sense in these fundamental ways [7] [p. 47].

Abner Shimony suggested calling this interpretation of the math or “formalism of quantum mechanics” as the *Literal Interpretation*.

These statements… may collectively be called “the Literal Interpretation” of quantum mechanics. This is the interpretation resulting from taking the formalism of quantum mechanics literally, as giving a representation of physical properties themselves, rather than of human knowledge of them, and by taking this representation to be complete [8] [pp. 6–7].

## 3. Results

### 3.1. The Lattice of Partitions

A *partition* π on a set $U=\{u_{1},\ldots ,u_{n}\}$ is a set of nonempty subsets or blocks $\pi =\{B_{1},\ldots ,B_{m}\}$ that are mutually disjoint and jointly exhaustive (their union is *U*). We stick to the finite case since our purpose is conceptual rather than obtaining mathematical generality. An equivalent definition, that prefigures the Hilbert space notion of a “direct-sum decomposition” of the space in terms of the eigenspaces of a Hermitian operator, is a set of nonempty subsets $\pi =\{B_{1},\ldots ,B_{m}\}$, such that every nonempty subset S⊆U can be uniquely represented as the union of a set of nonempty subsets of the $B_{j}$—in particular $S=\cup \{S\cap B_{j}\neq \emptyset :j=1,\ldots ,m\}$.

As the mathematical tool to describe distinctions versus indistinctions, a *distinction* or *dit* of π is an ordered pair of elements of *U* in different blocks of the partition, and the *ditset* dit(π) is the set of all the distinctions of π (also called an “apartness relation”). An *indistinction* or *indit* of π is an ordered pair of elements in the same block of the partition, and the *indit set* $\mathrm{indit}\left(\pi \right)=\cup_{j=1}^{m}B_{j}\times B_{j}$ is the set of all indits of π–which is the equivalence relation associated with π whose equivalence classes are the blocks of π.

The *partial order* on partition is usually defined as σ≾π (where $\sigma =\{C_{1},\ldots ,C_{m^{\prime }}\}$) if, for every $B_{j}\in \pi$, there is a $C_{j^{\prime }}\in \sigma$ such that $B_{j}\subseteq C_{j^{\prime }}$, but it is easier to just define it by σ≾π if dit(σ)⊆dit(π). The join (least upper bound) and meet (greatest lower bound) operations on partitions on *U* form the partition lattice Π(U). The most important operation for our purposes is the join operation where the *join* π∨σ is the partition on *U* whose blocks are the nonempty subsets $B_{j}\cap C_{j^{\prime }}$ for j=1,…,m and $j^{\prime }=1,\ldots ,m^{\prime }$. It could also be defined using ditsets, since dit(π∨σ)=dit(π)∪dit(σ).

The *meet* π∧σ of π and σ can be defined as the partition determined by the intersection of all the equivalence relations containing the equivalence relations corresponding to π and σ.

The partition lattice Π(U) also has a top and bottom. The top is the *discrete partition* $1_{U}=\left\{\left\{u_{1}\right\},\ldots ,\left\{u_{n}\right\}\right\}$ with only singleton blocks, which makes all possible distinctions, i.e., $\mathrm{dit}(1_{U})=U\times U-\Delta$ (where Δ is the diagonal of self-pairs $\left(u_{i},u_{i}\right)$). The bottom is the *indiscrete partition* or “The Blob”. Since $0_{U}$ is below all other partitions π on *U*, it is called “The Blob” because, as in the eponymous Hollywood movie, the Blob absorbs everything it meets, i.e., $0_{U}\wedge \pi =0_{U}$. The indiscrete partition $0_{U}=\left\{U\right\}$ has only one block *U* and it makes no distinctions, so $\mathrm{dit}(0_{U})=\emptyset$ and $\mathrm{indit}(0_{U})=U\times U$.

In terms of the old notions of matter (or substance) and form, the bottom of the partition lattice $0_{U}$ has all the substance but no form (no dits). The quantum notion of distinction-making change is prefigured by moving up the partition lattice as the substance is increasingly in-formed by distinctions until finally reaching the classical state $1_{U}$. As a metaphor for the Big Bang, in the beginning there was all the substance (energy) but with no distinctions, i.e., “perfect symmetry” [9], and then distinctions were introduced by symmetry-breaking to create (more definite) “its from dits.” That partition notion of becoming (indefinite to more definite) is particularly important for our purposes since it prefigures the notion of quantum (projective) measurement (von Neumann’s Type I processes) as well as quantum jumps.

**Figure 1:**
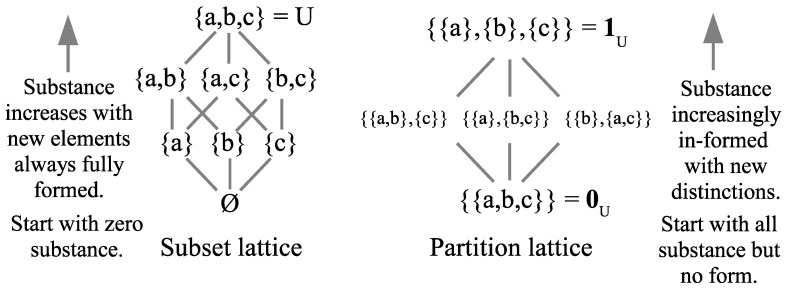
Two possible types of becoming given by two dual logical lattices.

The other logical notion of becoming or creating is illustrated by moving up the Boolean lattice of subsets. In the beginning, there was no substance (empty set) and then becoming takes place by the creation (ex nihilo) of “its” that are fully formed, as illustrated in Figure 1.

Metaphors might help to conceptualize the difference between the classical notion of change and quantum notion of becoming. In a police book of mug shots, the faces are fully formed and a metaphor for classical change is flipping through the fully definite or determinate mug shots. The quantum notion of becoming is like the police artist’s sketch pad where the indefinite outline of a face becomes more and more definite. Or, a white (superposition of all colors) painter’s canvas becomes a painting as the white superposition of colors “collapses” to take on definite colors. Since there are layers or levels of increasing definiteness as one moves up the lattice of partitions, another metaphor might be 3D printing where the printed object takes shape as the printer moves up to higher layers.

The mathematics for this transition from indefinite to more definite is the refining of partitions. The logical operation on partitions that results in a refinement is the join of partitions (i.e., the intersection of equivalence relations) which prefigures, at the quantum level, the Lüders mixture operation for projective measurement. The mathematics of QM is the extension of that partition math to complex Hilbert space.

### 3.2. The Logic of Partitions

The partition join and meet operations were known in the nineteenth century (e.g., Dedekind and Schröder). Ordinary logic is based on the Boolean logic of subsets of a universe set *U*; propositional logic is the special case of a one-element universe *U*. In subset logic, the formulas, in general, stand for subsets of some universe set *U*. Furthermore, subsets (or generally subobjects or ‘parts’) are category-theoretically dual to partitions (or generally quotient objects). “The dual notion (obtained by reversing the arrows) of ‘part’ is the notion of partition” [10] [p. 85]. Hence, one would naturally expect there to be a dual *logic* of partitions, where the formulas stand for partitions on some universe set *U*. However, that would require at least the operation of implication on partitions (corresponding to the Boolean conditional S⊃T), but no new operations on partitions were defined in the twentieth century. As acknowledged in a 2001 volume commemorating Gian-Carlo Rota: “the only operations on the family of equivalence relations fully studied, understood and deployed are the binary join ∨ and meet ∧ operations” [11] [p. 445]. Only in the current century was the implication operation σ⇒π on partitions (which turns the partition lattice Π(U) into an algebra) defined, along with general algorithms to turn subset logical operations into partition logical operations. The resulting logic of partitions cemented the notion of a partition as not just a mathematical concept of combinatorics but a logical concept ([1,12]).

There is a parallel development of subset logic and partition logic based on the dual connection between the elements, or “its”, of a subset and the distinctions, or “dits”, of a partition—which is summarized in Table 1. At the mathematical level, the dual lattices or logics are equally fundamental.

In subset logic, a formula is valid if, for any *U* ( |U|≥1) and any subsets of *U* substituted for the atomic variables, the formula evaluates by the logical subset operations to the top *U*. Similarly, in partition logic, a formula is valid if, for any *U* (|U|≥2) and any partitions on *U* substituted for the atomic variables, the formula evaluates by the logical partition operations to the top $1_{U}$. The partition validities are a proper subset of the Boolean subset validities but are neither contained in, nor contain, the valid formulas of intuitionistic logic (Heyting algebras).

### 3.3. Logical Information Theory: Logical Entropy

In their writings (and MIT lectures), Gian-Carlo Rota further developed the parallelism between subsets and partitions by considering their quantitative versions: “The lattice of partitions plays for information the role that the Boolean algebra of subsets plays for size or probability” [13] [p. 30]. Since the normalized size of a subset $\mathrm{Pr}\left(S\right)=\frac{\left|S\right|}{\left|U\right|}$ gives its Boole–Laplace finite probability, the “size” of a partition would play a similar role for information:

$$\frac{\mathrm{Information}}{\mathrm{Partitions}}\approx \frac{\mathrm{Probability}}{\mathrm{Subsets}}$$

Since “Probability is a measure on the Boolean algebra of events” that gives quantitatively the “intuitive idea of the size of a set”, we may ask by “analogy” for some measure “which will capture some property that will turn out to be for [partitions] what size is to a set” [14] [p. 67]. The duality (represented in Table 1) tells us that it is the number of dits in a partition that gives its size (maximum at the top and minimum at the bottom of the partition lattice) that is parallel to the number of ‘its’ in a subset (maximum at the top and minimum at the bottom of the subset lattice).

That is the reasoning that motivates the definition of the *logical entropy* of a partition as the normalized size of its ditset (equiprobable points):

$$h\left(\pi \right)=\frac{\left|\mathrm{dit}(\pi )\right|}{\left|U\times U\right|}=\frac{\left|U\times U|-|\mathrm{indit}(\pi )\right|}{\left|U\times U\right|}=1-\frac{\cup_{j}\left|B_{j}\times B_{j}\right|}{\left|U\times U\right|}=1-\sum_{j}{\left(\frac{|B_{j}|}{\left|U\right|}\right)}^{2}.$$

In general, if the points of $U=\{u_{1},\ldots ,u_{n}\}$ have general positive probabilities $p=\{p_{1},\ldots ,p_{n}\}$, then $\mathrm{Pr}\left(B_{j}\right)=\sum_{u_{i}\in B_{j}}p_{i}$, so that:

$$h\left(\pi \right)=1-\sum_{j=1}^{m}\mathrm{Pr}{\left(B_{j}\right)}^{2}=\sum_{j=1}^{m}\mathrm{Pr}\left(B_{j}\right)\left(1-\mathrm{Pr}\left(B_{j}\right)\right)=\sum_{j\neq j^{\prime }}\mathrm{Pr}\left(B_{j}\right)\mathrm{Pr}\left(B_{j^{\prime }}\right).$$

The parallelism carries through to the interpretation: $\mathrm{Pr}\left(S\right)=\sum_{u_{i}\in S}p_{i}$ is the one-draw probability of getting an ‘it’ of *S* and h(π) is the two-draw probability of getting a ‘dit’ of π.

The logical entropy h(π) is a (probability) measure in the sense of measure theory, i.e., h(π) is the product measure p×p on the ditset dit(π)⊆U×U. As a measure, the compound notions of joint, conditional, and mutual logical entropy satisfy the usual Venn diagram relationships.

The well-known Shannon entropy $H\left(\pi \right)=\sum_{j=1}^{m}\mathrm{Pr}\left(B_{j}\right)\log_{2}\left(\frac{1}{\mathrm{Pr}(B_{j})}\right)$ can also be interpreted in terms of partitions; it is the minimum average number of binary partitions (bits) it takes to distinguish the blocks of π. The Shannon entropy is not a measure in the sense of measure theory, but the compound notions of joint, conditional, and mutual Shannon entropy were defined so that they satisfy similar Venn-like pictures (but with no underlying set). That is possible because there is a non-linear but monotonic dit-to-bit transform, i.e., $1-\mathrm{Pr}\left(B_{j}\right)⇝\log_{2}\left(\frac{1}{\mathrm{Pr}(B_{j})}\right)$, that takes $h\left(\pi \right)=\sum_{j}\mathrm{Pr}\left(B_{j}\right)\left(1-\mathrm{Pr}\left(B_{j}\right)\right)$ to $H\left(\pi \right)=\sum_{j}\mathrm{Pr}\left(B_{j}\right)\log_{2}\left(\frac{1}{\mathrm{Pr}(B_{j})}\right)$ and which preserves Venn diagrams [2].

If a partition π is the inverse-image partition $\pi ={\left\{f^{-1}\left(r\right)\right\}}_{r\in f(U)}$ of a numerical attribute f:U→R, then h(π) is the two-draw probability of getting different *f*-values. The notion of logical entropy generalizes naturally to the notion of quantum logical entropy ([2,15]), where it gives the probability in two independent measurements of the same state by the same observable to which the result gives different eigenvalues.

### 3.4. Skeletonized Quantum States as Partitions

**Figure 2:**
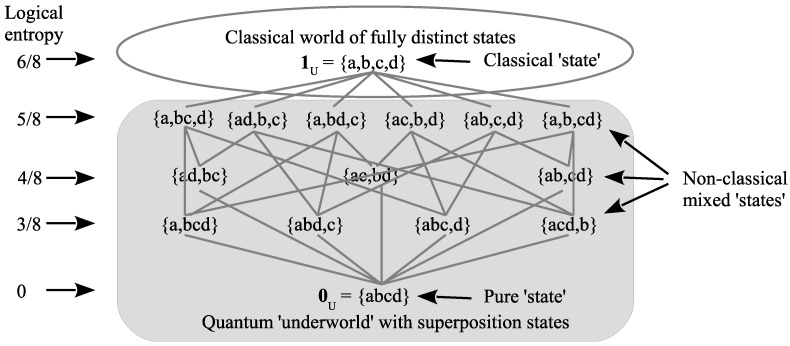
Skeletonized quantum states of a particle as the lattice of partitions.

There is a very simple way to skeletonize a quantum state to arrive at the corresponding set notion. Consider U={a,b,c,d} as both a set of distinct points (representing states of a particle) and also as an orthonormal basis for a four-dimensional Hilbert space. Then, a superposition state vector of the form (say) α|a〉+β|b〉 is *skeletonized* by deleting the complex scalars α and β, the Dirac kets, and the addition operation to yield just the *support*, the set {a,b} as a block in a partition, i.e., vector in QM math ⇝ support in partition math. A superposition of states skeletonizes to elements in the same block of a partition, i.e., superposition = equivalence or indistinction (which is non-trivial in the sense of not being a diagonal pair like (b,b)). Figure 2 is the lattice of partitions starting with a pure state (indiscrete partition), non-classical mixed states (i.e., mixed states that are more definite than the indiscrete partition but contain at least one superposition), and the classical mixed state with no superpositions. In Figure 2, we have used the partition shorthand of representing the partition {{a,b},{c,d}} as {ab,cd}, i.e., the innermost curly brackets are eliminated in favor of juxtaposition. The interpretation is that a,b,c, and *d* are distinct definite- or eigenstates of a particle according to some observable, where “particle” does not mean a classical (or Bohmian) particle, but an entity that can have different levels of objective indefiniteness (superpositions of the eigenstates such as a,b,c, or *d*) or the definiteness of an eigenstate. Perhaps “quantons”? [16]. The analysis is of standard von Neumann/Dirac quantum physics, not about quantum field theory where a particle might be analyzed as a “field quantum”.

Figure 2 is the Hasse diagram of the partition lattice on a four-element set, which means that each line between two partitions represents the partial order of refinement with no intermediate partitions. Then, the change from a partition to a more refined one is a “jump”. The skeletonized mixed states in the lattice of partitions of Figure 2 are all those that might be obtained by a “projective measurement” of the pure state {abcd}, i.e., by the partition join version of the Lüders mixture operation. All the lines or links in Figure 2 go from an indefinite state upward to a more definite state that is part of a join operation. A projective measurement turns a pure state into a mixed state, one part of which, e.g., an eigenstate in a maximal measurement, is realized according to its probability.

The set-level precursor of such a quantum jump from a superposition to an eigenstate is the “choice function” [17] [p. 60] which assigns to each nonempty subset (like a block in a partition) an element of the subset. That determination of an element is non-deterministic, except in the special case of a singleton set which is the precursor of the quantum measurement when the outcome has probability one, namely, when the state being measured is a single eigenstate of the observable being measured, as indicated in Table 2.

The indeterminism of the jump in the set-level choice function shows that those indeterminate aspects of QM math exist even prior to the introduction of probabilities.

The top discrete partition $1_{U}$ is the skeletal version of a classical mixed state, like randomly choosing a leaf in a four-leaf clover or randomly choosing a ‘letter’ in the four-letter genetic code U,C,A,G. One criterion of classical reality was the idea that it was fully definite or definite all-the-way-down, as in Leibniz’s Principle of Identity of Indistinguishables (PII) [18] [Fourth letter, 22] or Kant’s Principle of Complete Determination (*omnimoda determinatio*).

Every thing, however, as to its possibility, further stands under the principle of thoroughgoing determination; according to which, among all possible predicates of things, insofar as they are compared with their opposites, one must apply to it. [19] [p. B600]

Thus, two distinct things must have some predicate to distinguish them or, if there is no way to distinguish them, then they are the same thing. This principle of classicality characterizes the ‘classical’ state $1_{U}$:

$$\begin{array}{c} \mathrm{For}\mathrm{any}u,u^{\prime }\in U,\mathrm{if}\left(u,u^{\prime }\right)\in \mathrm{indit}\left(1_{U}\right),\mathrm{then}u=u^{\prime }\\ \mathrm{Partition}\mathrm{logic}\mathrm{version}\mathrm{of}\mathrm{Principle}\mathrm{of}\mathrm{Identity}\mathrm{of}\mathrm{Indistinguishables}. \end{array}$$

Any non-classical state in the skeletal representation is a partition π with a non-singleton ‘superposition’ block, e.g., {a,b} so (a,b)∈indit(π), but a≠b.

In contrast to a classical state, a quantum state is not fully definite, i.e., is not definite all-the-way-down. A state could even be maximally specified and still leave room for many particles (bosons) to be in that state.

In quantum mechanics, however, identical particles are truly indistinguishable. This is because we cannot specify more than a complete set of commuting observables for each of the particles; in particular, we cannot label the particle by coloring it blue [20] [p. 446].

In addition to PII, Leibniz had other metaphysical principles characteristic of the classical notion of reality. His Principle of Continuity was expressed by “*Natura non facit saltus*” (Nature does not make jumps) [21] [Bk. IV, chap. xvi] and his Principle of Sufficient Reason was expressed as the statement “that nothing happens without a reason why it should be so rather than otherwise” [18] [Second letter, 7]. All these classical principles are violated in the quantum world; PII is violated by superpositions of bosons, Continuity is violated by the quantum jumps, and Sufficient Reason is violated by the objective probabilities of QM.

This skeletal representation of quantum states is summarized in Table 3.

## 4. Superposition as Indefiniteness in the Quantum ‘Underworld’

**Figure 3:**
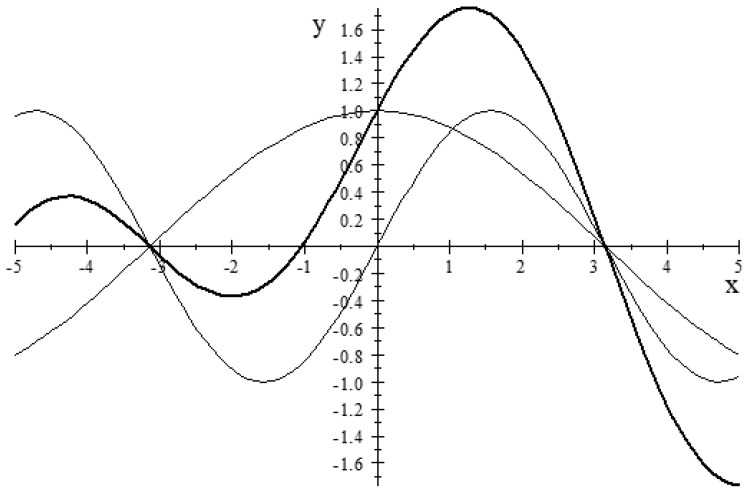
Misleading image of superposition in QM as sum of waves.

The biggest obstacle to understanding QM is the wave imagery, not to mention the name “wave mechanics”. That imagery interprets superposition ontologically as the sum of two waves to give another wave, as in Figure 3.

The mathematics of waves is always present, since any vector in a space over the complex numbers C automatically has wave imagery in the polar representation, characterized as having an amplitude and phase. However, the physical interpretation of waves is a misleading artifact or by-product of the use of complex numbers in the math of QM. The complex numbers are used because (among other reasons) they are algebraically complete so that the observable operators will have a complete set of eigenvectors [22] [p. 67, fn. 7]. In view of the century-long difficulties in interpreting QM as “wave mechanics”, Einstein’s statement: “The Lord is subtle, but not malicious”, may need to be nuanced. If not malicious, He is at least a trickster. It is a fact of the mathematics that the addition of vectors in a vector space over C can *always* be represented as the superposition of waves with interference effects.

The wave formalism offers a convenient mathematical representation of this latency, for not only can the mathematics of wave effects, like interference and diffraction, be expressed in terms of the addition of vectors (that is, their linear superposition; see [23] [Chap. 29.5]), but the converse, also holds [24] [p. 303]

On the partitional approach, a superposition (i.e., addition) of two definite- or eigenstates states {a} and {b} is a state {a,b} that is ontically indefinite or “blurred” between the two definite states. The partition approach requires the mental change-over from superposition-as-wave-addition to superposition-as-indefiniteness (since partitions are the math tool to describe indefiniteness and definiteness). Quantum reality is Indefinite World.

**Figure 4:**
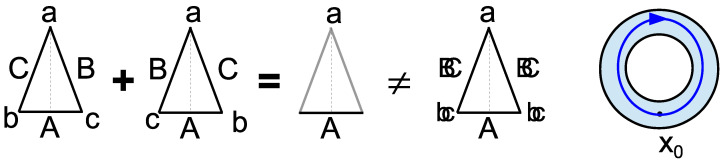
Imagery of superposition as indefiniteness.

For a pictorial image of the notion of superposition as creating indefiniteness or indistinction, the left side of Figure 4 gives the superposition of two isosceles triangles with labeled (i.e., distinguished) edges and vertices as the triangle that is indefinite on the edges and vertices where they differ and only definite where the two triangles are the same—and certainly not the triangle that is doubly definite (like a double-exposure photograph).

The fact that where superposed states have the same property is still definite (like vertex label *a*, the side label *A*) in the superposition is a little-noticed fact about quantum superpositions.

It follows from the linearity of the operators which represent observables of quantum mechanical systems that any measurable physical property which happens to be shared by all of the individual mathematical terms of some particular superposition (written down in any particular basis) will necessarily also be shared by the full superposition, considered as a single quantum-mechanical state, as well [25] [p. 234].

This means that the notion of superposition in QM is the flip-side of the notion of abstraction (e.g., in mathematics) [26]. Diederik Aerts and colleagues have extensively developed this connection [27,28,29]. In a superposition, the emphasis is on the indefiniteness resulting from where the elements of a set (i.e., a non-singleton equivalence class in an equivalence relation) differ like the vertex labels *b* and *c*, and the side labels *B* and *C* in Figure 4, while in abstraction, the emphasis is on the properties that are the same for the elements in the set (or equivalence class) like the vertex label *a* and side label *A*. It is like the flip-side of different viewpoints seeing the same glass as half-empty or as half-full.

In the older homotopy theory, the abstraction of a “homotopy type” was represented by a block in a partition, i.e., by an equivalence class. “Homotopy types are equivalence classes of spaces” under the equivalence relation “of deformation or homotopy” [30] [p. 4]. In the modern treatment [31], the equivalence class of, say, unit-interval-coordinatized paths going once around the ring clockwise (right side of Figure 4) is abstracted to an object having those common characteristics, but is not itself one of the differing paths in the equivalence class.

This insight that superposition as indefiniteness-on-differences is the flip-side of abstraction as definiteness-on-commonalities is, of course, unavailable when superposition is thought of as wave-addition.

Any quantum theorist who realizes that a superposition in the measurement basis does not have a definite value prior to the measurement already has a strong hint as to the nature of quantum reality. Some quantum theorists have taken the hint and envisioned a quantum world characterized by that indefiniteness where that “blurriness is an ontic feature” [32] [p. 163], where “the quantum mechanical unsharpness of measurable properties must be considered as an objective indeterminateness” [33] [p. 178], and where “inherent indefiniteness is a universal and objective property of matter” [34] [p. 202]. Thus, it is not a new idea that there is a quantum ‘underworld’ of indefinite superposition states ‘beneath’ the classical space-time world of definite states. That view was often expressed in the language of the quantum world of potentialities (or latencies) versus the classical world of definite actualities ([35,36]; [24] [Section 10.2]; [8,37]; [38] [Quantum Reality #8]; [39,40,41,42,43,44]).

Heisenberg [35] [p. 53]… used the term “potentiality” to characterize a property which is objectively indefinite, whose value when actualized is a matter of objective chance, and which is assigned a definite probability by an algorithm presupposing a definite mathematical structure of states and properties. Potentiality is a modality that is somehow intermediate between actuality and mere logical possibility. That properties can have this modality, and that states of physical systems are characterized partially by the potentialities they determine and not just by the catalogue of properties to which they assign definite values, are profound discoveries about the world, rather than about human knowledge [8] [p. 6].

Ruth Kastner uses the imagery of an iceberg [43] [p. 3] with the classical world above water and the quantum world beneath the water—an imagery that is filled out by Figure 2 imagery of the partition lattice as the skeletonized classical and quantum states. The language of “potentialities” or the Aristotelian notion of “potentia” is not very felicitous since they are taken to be realities, not mere possibilities. Hence, some prefer the language of “latencies” (e.g., Henry Margenau and R. I. G. Hughes), but, in both the cases of “potentialities” and “latencies”, the key idea is objective indefiniteness.

The historical reference should perhaps be dismissed, since quantum mechanical potentiality is completely devoid of teleological significance, which is central to Aristotle’s conception. What it has in common with Aristotle’s conception is the indefinite character of certain properties of the system. [45] [pp. 313–314]

Furthermore, Margenau notes that the measurement of observables “forces them out of indiscriminacy or latency” [36] [p. 10]—which indicates that Margenau also interprets “latency” in terms of indeterminacy or indefiniteness. Kastner also considers the indeterminacy of values as a key characteristic of the real potentia [43] [p. 3]. “Although the quantum possibilities represent properties that might be observed, they are not determinate (that is, they are not well defined)” [46] [p. 27]. In short, it is not real potentialities versus real actualities, but real (i.e., objective) indefiniteness versus real definiteness. Those who acknowledge objective indefiniteness from a Whiteheadian approach might be mentioned, e.g., [47,48,49]. There is only one type of reality and it occurs in varying degrees of indefiniteness, as represented in skeletal form by the partition lattice (Figure 2). Partitions (or equivalence relations) do not contain any different modalities like potentiality and actuality, only different degrees of indefinite to definite realities (so Occam’s Razor could be applied to Aristotle’s two modalities).

The main brain-twisting reconceptualization required to understand quantum reality is as follows:

- Where the classical notion of fully definite reality is replaced by the idea of indefiniteness at the quantum level (not definite all-the-way-down),
- Where the state-reduction jump from an indefinite state to a more definite state is due to making distinctions (“more-definite its from dits”),
- Where change with no distinctions stays at the same level of indefiniteness (unitary evolution).

Classical mechanics has only one type of change, definite to definite at the level of fully definite classical reality.

After a century of the philosophically fruitless conceptualization of QM states in terms of wave functions, is there another equivalent mathematical tool to represent a quantum state that makes the indefiniteness obvious? Yes, it is the density matrix. We will gradually approach density matrices in order to show the origin of the Born rule in the concept of superposition.

### 4.1. Superposition and the Born Rule

“Where does the Born rule come from?” [22] [p. 92]. To answer this question, we make the minimal extension of standard probability theory to introduce the notion of a superposition subset or event as the ‘blurred’ or ‘blobbed together’ version of a discrete subset or event. Consider the outcome set $U=\left\{a,b,c\right\}$ of the three labeled vertices of an equilateral triangle ${\overset{a}{{\;}_{b}▵}}_{c}$. The usual discrete event consisting of those three distinguished outcomes could be represented as a column vector $|U\rangle =$$\left[\begin{array}{c} 1\\ 1\\ 1 \end{array}\right]$. However, how can we represent the superposition event that blurs those three vertices together? Visually, think of the triangle ▵, or perhaps ${\overset{?}{{\;}_{?}▵}}_{?}$, with unlabeled, i.e., undistinguished, vertices.

To distinguish the discrete set from the superposition set, we need to move up one dimension to consider a 3×3 matrix. An *incidence matrix* $\mathrm{In}\left(R\right)$ of a binary relation R⊆U×U has entries 1 or 0 depending on whether the (row, column) ordered pair is in the relation *R* or not, e.g., $\mathrm{In}{\left(R\right)}_{a,b}=1$ if $\left(a,b\right)\in R$, otherwise 0. The discrete set *U* is represented by the incidence matrix for the diagonal binary relation $\Delta U=\left\{\left(a,a\right),\left(b,b\right),\left(c,c\right)\right\}$, so that incidence matrix is:

$$\mathrm{In}\left(\Delta U\right)=\left[\begin{array}{ccc} 1 & 0 & 0\\ 0 & 1 & 0\\ 0 & 0 & 1 \end{array}\right].$$

The superposition set, denoted ΣU, where the elements of *U* are blobbed together is represented by the incidence matrix for the universal relation U×U:

$$\mathrm{In}\left(U\times U\right)=\left[\begin{array}{ccc} 1 & 1 & 1\\ 1 & 1 & 1\\ 1 & 1 & 1 \end{array}\right].$$

The off-diagonal elements represent which elements are blobbed or ‘cohered’ together in the superposition. The only (nonempty) events that are the same in their discrete version and their superposition version are the singleton events and, accordingly, their incidence matrices are the same.

It is important to note that the introduction of the two-dimensional matrix (as opposed to the one-dimensional vector) to represent superposition events allows the representation by outer products (where the superscript *t* is transposed and ${|U\rangle }^{t}=\langle U|$), e.g.,

$$\left[\begin{array}{c} 1\\ 1\\ 1 \end{array}\right]{\left[\begin{array}{c} 1\\ 1\\ 1 \end{array}\right]}^{t}=|U\rangle \langle U|=\mathrm{In}\left(U\times U\right)=\left[\begin{array}{ccc} 1 & 1 & 1\\ 1 & 1 & 1\\ 1 & 1 & 1 \end{array}\right].$$

In a partition, the elements inside a block or equivalence class are blobbed together. For instance, the partition $\pi =\left\{\left\{a,c\right\},\left\{b\right\}\right\}$ is represented by the incidence matrix:

$$\begin{array}{c} \mathrm{In}\left(\pi \right)=|\left\{a,c\right\}\rangle \langle \left\{a,c\right\}|+|\left\{b\right\}\rangle \langle \left\{b\right\}|=\left[\begin{array}{c} 1\\ 0\\ 1 \end{array}\right]\left[\begin{array}{ccc} 1 & 0 & 1 \end{array}\right]+\left[\begin{array}{c} 0\\ 1\\ 0 \end{array}\right]\left[\begin{array}{ccc} 0 & 1 & 0 \end{array}\right]\\ =\left[\begin{array}{ccc} 1 & 0 & 1\\ 0 & 0 & 0\\ 1 & 0 & 1 \end{array}\right]+\left[\begin{array}{ccc} 0 & 0 & 0\\ 0 & 1 & 0\\ 0 & 0 & 0 \end{array}\right]=\left[\begin{array}{ccc} 1 & 0 & 1\\ 0 & 1 & 0\\ 1 & 0 & 1 \end{array}\right]. \end{array}$$

To visualize the partition π, one might think of an equilateral triangle ${\;}_{b}▵$, or perhaps ${\overset{?}{{\;}_{b}▵}}_{?}$, with one vertex definitely labeled *b*, but with the other two vertices undistinguished, i.e., which is the superposition ${\overset{?}{{\;}_{b}▵}}_{?}={\overset{a}{{\;}_{b}▵}}_{c}+{\overset{c}{{\;}_{b}▵}}_{a}$ that is definite on where the superposed states are the same and is indefinite on where they differ. The five non-zero elements of $\mathrm{In}\left(\pi \right)$ represent the five ordered pairs in the equivalence relation $\mathrm{indit}\left(\pi \right)=\left\{\left(a,a\right),\left(b,b\right),\left(c,c\right),\left(a,c\right),\left(c,a\right)\right\}$ and the {(a,b),(c,b),(b,a),(b,c)}=dit(π) corresponds to the four zeros in the matrix.

The inner product allows the specification of a component of a vector, e.g., $\langle \left\{a\right\}|\left\{a,c\right\}\rangle =1$ and $\langle \left\{b\right\}|\left\{a,c\right\}\rangle =0$. At this stage, the components of the vectors are idempotent, so ${\langle \left\{a\right\}|\left\{a,c\right\}\rangle }^{2}=1$ and ${\langle \left\{b\right\}|\left\{a,c\right\}\rangle }^{2}=0$.

Finally, probabilities are introduced by initially assuming all outcomes are equiprobable. Then, the incidence matrices are turned into density matrices by dividing through by the trace so, for instance, the density matrix for π is:

$$\rho \left(\pi \right)=\frac{\mathrm{In}(\pi )}{tr[\mathrm{In}(\pi )]}=\left[\begin{array}{ccc} \frac{1}{3} & 0 & \frac{1}{3}\\ 0 & \frac{1}{3} & 0\\ \frac{1}{3} & 0 & \frac{1}{3} \end{array}\right]$$

where, again, the non-zero entries represent the indistinctions of π and the zeros represent the distinctions.

A density matrix ρ is *pure* if $\mathrm{tr}\left[\rho^{2}\right]=1$, otherwise *mixed*. The pure density matrices can again be obtained as outer products. For instance, the density matrix for the superposition state $\Sigma \left\{a,c\right\}$ (non-singleton blocks in partitions are always interpreted as superpositions) is:

$$\rho \left(\Sigma \left\{a,c\right\}\right)=\frac{1}{\sqrt{\left|\left\{a,c\right\}\right|}}|\left\{a,c\right\}\rangle \langle \left\{a,c\right\}|\frac{1}{\sqrt{\left|\left\{a,c\right\}\right|}}=\left[\begin{array}{c} \frac{1}{\sqrt{2}}\\ 0\\ \frac{1}{\sqrt{2}} \end{array}\right]\left[\begin{array}{ccc} \frac{1}{\sqrt{2}} & 0 & \frac{1}{\sqrt{2}} \end{array}\right]=\left[\begin{array}{ccc} \frac{1}{2} & 0 & \frac{1}{2}\\ 0 & 0 & 0\\ \frac{1}{2} & 0 & \frac{1}{2} \end{array}\right].$$

Given the event $\Sigma \left\{a,c\right\}$ (or the discrete event $\left\{a,c\right\}$, for that matter) the conditional probability of $\left\{a\right\}$ is $\frac{1}{2}$ and that is obtained as the:

$$\begin{array}{c} {\langle \left\{a\right\}|\frac{1}{\sqrt{2}}\left\{a,c\right\}\rangle }^{2}=\frac{1}{2}\\ \mathrm{Born}\mathrm{rule} \end{array}$$

which could also be obtained as the trace of the projection matrix to $\left\{a\right\}$ times the density matrix $\rho \left(\Sigma \left\{a,c\right\}\right)$, i.e.,

$$\mathrm{tr}\left[\left[\begin{array}{ccc} 1 & 0 & 0\\ 0 & 0 & 0\\ 0 & 0 & 0 \end{array}\right]\left[\begin{array}{ccc} \frac{1}{2} & 0 & \frac{1}{2}\\ 0 & 0 & 0\\ \frac{1}{2} & 0 & \frac{1}{2} \end{array}\right]\right]=\mathrm{tr}\left[\begin{array}{ccc} \frac{1}{2} & 0 & \frac{1}{2}\\ 0 & 0 & 0\\ 0 & 0 & 0 \end{array}\right]=\frac{1}{2}.$$

No quantum *physics* was used in this subsection. We only made the minimal extension to classical finite probability theory to introduce the purely mathematical notion of superposition events in addition to the usual discrete events. The mathematical treatment of the superposition events, as opposed to the classical discrete events, required moving to two-dimensional matrices, first incidence matrices and then density matrices, and then the probabilities emerged by the Born rule. In that sense, the non-classical Born rule is a feature of the key non-classical concept of superposition. This thesis, “the Born rule is a feature of superposition”, is further corroborated by considering the limit case of the measurement of a completely decomposed mixed state with a diagonal density matrix. Then, there is no superposition, each state is specified by its probability, and there are no amplitudes to be squared in the Born rule, so we are back in the domain of the “no superposition & no Born rule” classical probability theory. The *math* of superposition brings on the Born rule.

We turn now to density matrices with general probability distributions.

### 4.2. Quantum States

To demonstrate our thesis that the math of QM is the Hilbert space version of the math of partitions, we need to first focus on the three main concepts in the math of QM: (1) the quantum state, (2) the quantum observable, and (3) the quantum (always projective) measurement.

A quantum state can be presented either as a state vector or as a density matrix [50]. The density matrix approach best displays the relevant information for the partitional interpretation in terms of indefiniteness. Hence, we start by transferring the structure of a partition π into its density matrix form ρ(π). The initial data is a partition $\pi =\{B_{1},\ldots ,B_{m}\}$ on *U* with (positive) point probabilities $p=(p_{1},\ldots ,p_{n})$. For each $B_{j}\in \pi$, we define $|b_{j}\rangle$ as the column vector with the $i^{th}$ entry being $\sqrt{p_{i}/\mathrm{Pr}\left(B_{j}\right)}$ if $u_{i}\in B_{j}$, else 0 so that $\langle b_{k}|b_{j}\rangle =\delta_{jk}$. We form the projection density matrix $\rho \left(B_{j}\right)=|b_{j}\rangle \langle b_{j}|$ with the i,k-entry being $\rho {\left(B_{j}\right)}_{ik}=\frac{\sqrt{p_{i}p_{k}}}{\mathrm{Pr}(B_{j})}$ if $u_{i},u_{k}\in B_{j}$, else 0. It should be noted how the indefiniteness interpretation of superposition comes out in the mathematics. The set-level superposition of $u_{i}$ and $u_{k}$ in $B_{j}$ implies the non-zero off-diagonal element in $\rho (B_{j})$ indicating indefiniteness.

These density matrices $\rho (B_{j})$ of pure superposition states also show the origin of the Born rule (as indicated above). Taking $B_{j}=S$, an arbitrary nonempty (superposition) subset of *U*, the ith entry of $|s\rangle$ is $\langle u_{i}|s\rangle =\sqrt{\frac{p_{i}}{\mathrm{Pr}\left(S\right)}}\chi_{S}\left(u_{i}\right)$. Then, the probability of $u_{i}$ conditioned on *S* is given by the Born rule: ${\langle u_{i}|s\rangle }^{2}=\frac{p_{i}}{\mathrm{Pr}\left(S\right)}\chi_{S}\left(u_{i}\right)=\mathrm{Pr}\left(u_{i}|S\right)$ (in QM math over C, it would be absolute square).

For the partition π, the density matrix ρ(π) is the probability sum of these projectors:

$$\rho \left(\pi \right)=\sum_{j=1}^{m}\mathrm{Pr}\left(B_{j}\right)|b_{j}\rangle \langle b_{j}|.$$

Then, it is easily checked that $\rho {\left(\pi \right)}_{ik}=\sqrt{p_{i}p_{k}}$ if $\left(u_{i},u_{k}\right)\in \mathrm{indit}\left(\pi \right)$, else 0. Thus, the non-zero entries in ρ(π) represent the indits of π and the zero entries represent the distinctions of π. A density matrix is not only Hermitian, but positive, so its eigenvalues are non-negative real numbers $\lambda_{i}$ which sum to 1, i.e., $\sum_{i=1}^{n}\lambda_{i}=1$. In the case of ρ(π), these $\lambda_{i}$ are *m* non-zero eigenvalues $\mathrm{Pr}(B_{j})$ with the remaining n−m eigenvalues of 0.

For the classical state $1_{U}$, its density matrix $\rho (1_{U})$ is a diagonal matrix with the point probabilities along the diagonal, e.g., “the statistical mixture describing the state of a classical dice before the outcome of the throw” [51] [p. 176]. Thus, the non-classical states in the skeletal representation are the ones where ρ(π) has non-zero off-diagonal elements indicating the ‘amplitudes’ $\sqrt{p_{i}p_{k}}$ of the corresponding diagonal states ($u_{i}$ and $u_{k}$) blobbing or cohering together in a superposition. Since superposition states are the key non-classical states, it is these non-zero off-diagonal “coherences” [52] [p. 303] that account for the non-classical interference effects in the Hilbert space version.

The off-diagonal terms of a density matrix … are often called quantum coherences because they are responsible for the interference effects typical of quantum mechanics that are absent in classical dynamics [51] [p. 177].

In the full non-skeletal Hilbert space case of a density matrix ρ, it has a spectral decomposition $\rho =\sum_{i=1}^{n}\lambda_{i}|u_{i}\rangle \langle u_{i}|$ with an orthonormal basis $\{|u_{i}\rangle {\}}_{i=1}^{n}$ so $\langle u_{i^{\prime }}|u_{i}\rangle =\delta_{ii^{\prime }}$ and where the non-negative eigenvalues $\lambda_{i}$ sum to 1. Thus, for the concept of a quantum state, we have the skeletal set-level presentation of a partition and the corresponding Hilbert space version of that partition math as summarized in Table 4.

### 4.3. Quantum Observables

We have seen how the notion of a quantum state was prefigured at the set level by (i.e., has the set-level precursor of) a partition on a set with point probabilities. In a similar manner, the notion of a quantum observable is prefigured at the set level by the inverse-image partition ${\left\{f^{-1}\left(r\right)\right\}}_{r\in f(U)}$ of a real-value numerical attribute f:U→R.

In the folklore of mathematics, there is a semi-algorithmic procedure to connect set concepts with the corresponding vector (Hilbert) space concepts. We will call it a “yoga” [53] [p. 251], the Yoga of Linearization:

$$\begin{array}{c} \mathrm{For}\mathrm{any}\mathrm{given}\mathrm{set-concept},\mathrm{apply}\mathrm{it}\mathrm{to}a\mathrm{basis}\mathrm{set}\mathrm{of}a\mathrm{vector}\mathrm{space}\\ \mathrm{and}\mathrm{whatever}\mathrm{is}\mathrm{linearly}\mathrm{generated}\mathrm{is}\mathrm{the}\mathrm{corresponding}\mathrm{vector}\mathrm{space}\mathrm{concept}.\\ The\;Yoga\;of\;Liniearization \end{array}$$

To apply the Yoga, we first take *U* as just a universal set and consider some set-based concept, and then we consider *U* as a basis set (e.g., an ON basis of a Hilbert space) of a vector space *V* and see what is linearly generated. The Yoga is mathematically based on the free vector space functor that takes a set in the category of sets to the free vector space $k^{U}=V$ in the category of vector spaces over a given field k, which we take to be C in the case of QM. A subset *S* of a basis set *U* generates a subspace [S] of the space *V*. The cardinality of the subset gives the dimension of the subspace. A real-valued numerical attribute f:U→R defines a Hermitian operator F:V→V (where V=[U]) by defining *F* on the basis set *U* as Fu=f(u)u or, using the Dirac notation, F|u〉=f(u)|u〉.

To better analyze the numerical attribute, let f↾S=rS stand for “the value of *f* on the subset *S* is *r*”. That is the set-level version of the eigenvalue/eigenvector equation F|u〉=r|u〉. Hence, we see that the set version of an eigenvector is a constant set *S* of *f* and the set version of an eigenvalue of an eigenvector is the constant value *r* on a constant set *S*. A characteristic function $\chi_{S}:U\to \left\{0,1\right\}\subseteq R$ has only two constant sets $S=\chi_{S}^{-1}\left(1\right)$ and $S^{c}=U-S=\chi_{S}^{-1}\left(0\right)$. The Yoga yields the corresponding vector space notion which is a projection operator $P_{\left[S\right]}$, which is defined by $P_{\left[S\right]}|u\rangle =|u\rangle$ if u∈S, else 0 (zero vector) which has the eigenvalues of 0 and 1.

A Hermitian (or self-adjoint) operator *F* in QM has spectral decomposition $F=\sum_{\lambda_{i}}\lambda_{i}P_{V_{i}}$ where the sum is over the real eigenvalues $\lambda_{i}$ and the projections $P_{V_{i}}$ to their eigenspaces. Bearing in mind the correlation given by the Yoga, we can define the ‘spectral decomposition’ of the numerical attribute f:U→R as $f=\sum_{r\in f(U)}r\chi_{f^{-1}\left(r\right)}$. Starting at the quantum level with a Hermitian operator F:V→V and a basis set *U* of eigenvectors of *F*, then going backwards to the set level, *f* is obtained as the eigenvalue function f:U→R.

An important application of the Yoga is to the notion of a set partition $\pi ={\left\{f^{-1}\left(r\right)\right\}}_{r\in f(U)}$ as the inverse-image of a numerical attribute. Applied to the basis set $\{|u_{i}\rangle {\}}_{i=1}^{n}$ used to define *F* by $F|u_{i}\rangle =f\left(u_{i}\right)|u_{i}\rangle$, each block $f^{-1}\left(r\right)$ of π generates the eigenspace of eigenvectors for the eigenvalue *r*. These eigenspaces ${\left\{V_{r}\right\}}_{r\in f(U)}$ form a *direct-sum decomposition* (DSD) of *V*, i.e., $V={⊕}_{r\in f(U)}V_{r}$, where a DSD is defined as a set of non-zero subspaces ${\left\{V_{r}\right\}}_{r\in f(U)}$ such that every non-zero vector v∈V has a unique representation as a sum $v=\sum_{r\in f(U)}v_{r}$ of non-zero vectors $v_{r}\in V_{r}$. Since subsets linearize to subspaces, the logic of subsets leads to the Birkhoff–von Neumann quantum logic of subspaces [54]. Since partitions linearize to direct-sum decompositions, the logic of partitions leads to the quantum logic of DSDs [55].

It was noted previously that a set partition $\pi =\{B_{1},\ldots ,B_{m}\}$ has a similar definition since every nonempty subset *S* has a unique representation as the union of subsets of the ${\left\{B_{j}\right\}}_{j=1}^{m}$. If the union of the $B_{j}$s was not all of *U*, then $U-\cup_{j=1}^{m}B_{j}$ would have no representation, and if $S=B_{j}\cap B_{j^{\prime }}\neq \emptyset$, then *S* has two representations as subsets of the $B_{j}$s. Moreover, S∩():℘(U)→℘(U) is a projection operator on the power set ℘(U) that takes any subset T∈℘(U) to S∩T∈℘(U). Thus, we have $\cup_{r\in f(U)}\left(f^{-1}\left(r\right)\cap \left(\right)\right)=I:℘\left(U\right)\to ℘\left(U\right)$ whose quantum version is resolution of unity $\sum_{r\in f(U)}P_{V_{r}}=I:V\to V$. Furthermore, lastly, we might apply the Yoga to the Cartesian product $U\times U^{\prime }$ where *U* and $U^{\prime }$ are basis sets for *V* and $V^{\prime }$. Then, the ordered pairs $\left(u,u^{\prime }\right)\in U\times U^{\prime }$ (bi)linearly generate the tensor product $V⊗V^{\prime }$, where the ordered pair $\left(u,u^{\prime }\right)$ is customarily written as $u⊗u^{\prime }$ or $\left|u\rangle ⊗\right|u^{\prime }\rangle$.

These results of the Yoga of Linearization are summarized in the translation dictionary of Table 5, further showing how the math of QM is the Hilbert space version of partition math at the level of sets.

### 4.4. Quantum Measurement

The third basic concept to be analyzed is quantum measurement (always projective)—the quantum notion of becoming, i.e., change going from a level of indefiniteness to a level of more definiteness. In the more general case of positive-operator valued measurement, the change is still from indefinite or indeterminate to “less indeterminate” [41] [p. 120], so we will forego the additional complication and stick to projective measurement. The connection between the set-level notion of measurement and the quantum level is the Lüders mixture operation ([51] [p. 279] and [56]) that can be applied at both levels. At the set level, we have the skeletal state represented by a density matrix ρ(π) and we have an ‘observable’ or real-value numerical attribute, say, g:U→R whose inverse-image is the partition $\sigma =g^{-1}={\left\{g^{-1}\left(r\right)\right\}}_{r\in g(U)}$. The Lüders mixture operation applies the ‘observable’ to the density matrix ρ(π) to arrive at the post-measurement density matrix $\hat{\rho }\left(\pi \right)$. The operation uses the n×n projection matrices for the blocks $g^{-1}\left(r\right)$ of σ which are diagonal matrices $P_{g^{-1}\left(r\right)}$ whose diagonal elements are the values of the characteristic function $\chi_{g^{-1}\left(r\right)}$. Then, the post-measurement density matrix is:

$$\begin{array}{c} \hat{\rho }\left(\pi \right)=\sum_{r\in g(U)}P_{g^{-1}\left(r\right)}\rho \left(\pi \right)P_{g^{-1}\left(r\right)}\\ \mathrm{Partition}\mathrm{version}\mathrm{of}\mathrm{Lüders}\mathrm{mixture}\mathrm{operation}. \end{array}$$

Then, it is an easy result:

**Theorem** **1.** *$\hat{\rho }\left(\pi \right)=\rho \left(\pi \vee g^{-1}\right)$.*

*Thus, the set version of quantum-level projective measurement in the math of QM is the partition join operation where dit(π∨σ)=dit(π)∪dit(σ) and, by DeMorgan’s Law, indit(π∨σ)=indit(π)∩indit(σ) since the join of partitions operation can also be viewed as the intersection of equivalence relations. The non-classical quantum notion of becoming is the jump from π to the less-indefinite mixed state $\pi \vee g^{-1}$, and then to one of the states in the mixture according to its probability.*

**Example** **1.** *Let π={{a},{b,c}} with probabilities $\mathrm{Pr}\left(\left\{a\right\}\right)=\frac{1}{3}$, $\mathrm{Pr}\left(\left\{b\right\}\right)=\frac{1}{4}$, and $\mathrm{Pr}\left(\left\{c\right\}\right)=\frac{5}{12}$ in U={a,b,c} and σ={{a,b},{c}}, so $\sigma =g^{-1}$ for any g:U→R that assigns the same g-value to a and b with a different value for c. Then, the density matrix for π and the projections matrices for the blocks of σ are:*

$$\rho \left(\pi \right)=\left[\begin{array}{ccc} \frac{1}{3} & 0 & 0\\ 0 & \frac{1}{4} & \frac{\sqrt{5}}{4\sqrt{3}}\\ 0 & \frac{\sqrt{5}}{4\sqrt{3}} & \frac{5}{12} \end{array}\right],P_{\left\{a,b\right\}}=\left[\begin{array}{ccc} 1 & 0 & 0\\ 0 & 1 & 0\\ 0 & 0 & 0 \end{array}\right],and\;P_{\left\{c\right\}}=\left[\begin{array}{ccc} 0 & 0 & 0\\ 0 & 0 & 0\\ 0 & 0 & 1 \end{array}\right].$$

*The Lüders mixture operation is:*

$$\begin{array}{c} \hat{\rho }\left(\pi \right)=P_{\left\{a,b\right\}}\rho \left(\pi \right)P_{\left\{a,b\right\}}+P_{\left\{c\right\}}\rho \left(\pi \right)P_{\left\{c\right\}}\\ =\left[\begin{array}{ccc} 1 & 0 & 0\\ 0 & 1 & 0\\ 0 & 0 & 0 \end{array}\right]\left[\begin{array}{ccc} \frac{1}{3} & 0 & 0\\ 0 & \frac{1}{4} & \frac{\sqrt{5}}{4\sqrt{3}}\\ 0 & \frac{\sqrt{5}}{4\sqrt{3}} & \frac{5}{12} \end{array}\right]\left[\begin{array}{ccc} 1 & 0 & 0\\ 0 & 1 & 0\\ 0 & 0 & 0 \end{array}\right]\\ +\left[\begin{array}{ccc} 0 & 0 & 0\\ 0 & 0 & 0\\ 0 & 0 & 1 \end{array}\right]\left[\begin{array}{ccc} \frac{1}{3} & 0 & 0\\ 0 & \frac{1}{4} & \frac{\sqrt{5}}{4\sqrt{3}}\\ 0 & \frac{\sqrt{5}}{4\sqrt{3}} & \frac{5}{12} \end{array}\right]\left[\begin{array}{ccc} 0 & 0 & 0\\ 0 & 0 & 0\\ 0 & 0 & 1 \end{array}\right]\\ =\left[\begin{array}{ccc} \frac{1}{3} & 0 & 0\\ 0 & \frac{1}{4} & 0\\ 0 & 0 & 0 \end{array}\right]+\left[\begin{array}{ccc} 0 & 0 & 0\\ 0 & 0 & 0\\ 0 & 0 & \frac{5}{12} \end{array}\right]=\left[\begin{array}{ccc} \frac{1}{3} & 0 & 0\\ 0 & \frac{1}{4} & 0\\ 0 & 0 & \frac{5}{12} \end{array}\right]. \end{array}$$

**Figure 5:**
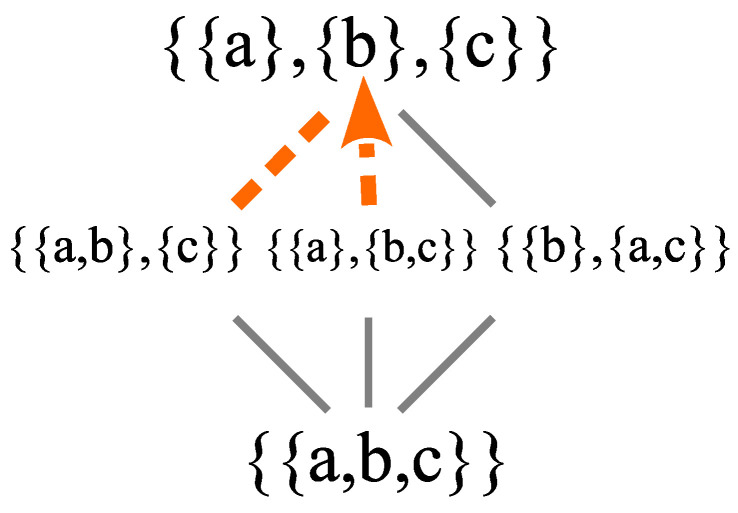
Partition lattice with join π∨σ={{a},{b,c}}∨{{a,b},{c}}={{a},{b},{c}}.

Since $\pi \vee \sigma =\left\{\left\{a\right\},\left\{b\right\},\left\{c\right\}\right\}=1_{U}$, we see that the post-measurement density matrix is $\hat{\rho }\left(\pi \right)=\rho \left(\pi \vee \sigma \right)=\rho \left(1_{U}\right)$. Thus, the superposition of {b} and {c} in π got distinguished since *b* and *c* had different *g*-values. The new distinctions in dit(σ)−dit(π) are (b,c) (along with (c,b)) and those were the non-zero off-diagonal elements (coherences) of ρ(π) that got zeroed (distinguished or decohered) in ρ(π∨σ). Figure 5 shows the join of {{a},{b,c}} and {{a,b},{c}} is their least upper bound $1_{U}=\left\{\left\{a\right\},\left\{b\right\},\left\{c\right\}\right\}$.

In the math of the partition join, the partitions are symmetrical. However, the Lüders mixture operation is not symmetric even through the result is the join. One partition (π={{a},{b,c}}) represents the state being measured and the other partition (σ={{a,b},{c}}) represents the observable. Since the join in Figure 5 is the discrete partition, the measurement represented by the join is maximal. The arrow in Figure 5 represents the jump from a less-refined mixed state π to the maximal state $1_{U}$.

The Lüders mixture is not the end of the measurement process. The measurement returns one of the *g*-values, say the (degenerate) one for {a,b}. Then, the Lüders Rule [24] [Appendix B] gives the final density matrix, which is the corresponding term in the Lüders mixture sum, e.g., $P_{\left\{a,b\right\}}\rho \left(\pi \right)P_{\left\{a,b\right\}}$, normalized so the final density matrix is the (in this case, classical) mixed state:

$$\frac{P_{\left\{a,b\right\}}\rho \left(\pi \right)P_{\left\{a,b\right\}}}{\mathrm{tr}[P_{\left\{a,b\right\}}\rho \left(\pi \right)P_{\left\{a,b\right\}}]}=\left[\begin{array}{ccc} \frac{1}{3} & 0 & 0\\ 0 & \frac{1}{4} & 0\\ 0 & 0 & 0 \end{array}\right]\frac{1}{7/12}=\left[\begin{array}{ccc} \frac{4}{7} & 0 & 0\\ 0 & \frac{3}{7} & 0\\ 0 & 0 & 0 \end{array}\right].$$

In the general Hilbert space case, the Hermitian operator *G* is given by its DSD of eigenspaces ${\left\{V_{r}\right\}}_{r\in g(U)}$ (where g:U→R is the eigenvalue function assigning the appropriate real eigenvalue to each vector in an ON basis *U* of eigenvectors of *G*). The state being measured is given by density matrix ρ expressed in the ON basis *U*, and the Lüders mixture operation uses the projection matrices $P_{V_{r}}$ to the eigenspaces of *G* to determine the post-measurement density matrix $\hat{\rho }$. The Hilbert space version of the set operation is:

$$\begin{array}{c} \hat{\rho }=\sum_{r\in g(U)}P_{V_{r}}\rho P_{V_{r}}\\ \mathrm{Hilbert}\mathrm{space}\mathrm{version}\mathrm{of}\mathrm{Lüders}\mathrm{mixture}\mathrm{operation}. \end{array}$$

We saw previously how the notion of logical entropy (and its quantum counterpart) was based on the notion of a quantitative measure of distinctions of a partition. Hence, logical entropy is the *natural* notion to measure the changes in a density matrix under a measurement. For instance, in the above example where π={{a},{b,c}}, the block probabilities are $\mathrm{Pr}\left(\left\{a\right\}\right)=\frac{1}{3}$ and $\mathrm{Pr}\left(\left\{b,c\right\}\right)=\frac{2}{3}$, and the logical entropy is $h\left(\pi \right)=1-\mathrm{Pr}{\left(\left\{a\right\}\right)}^{2}-\mathrm{Pr}{\left(\left\{b,c\right\}\right)}^{2}=1-\frac{1}{9}-\frac{4}{4}=\frac{4}{9}$. When the partition π is represented as a density matrix ρ(π), then the logical entropy could also be computed as follows where the density matrix formula of logical entropy has been called “mixedness” [57] [p. 5]:

$$h\left(\pi \right)=h\left(\rho \left(\pi \right)\right)=1-\mathrm{tr}\left[\rho {\left(\pi \right)}^{2}\right]=1-\mathrm{tr}\left[\begin{array}{ccc} \frac{1}{9} & 0 & 0\\ 0 & \frac{1}{6} & \frac{\sqrt{5}}{4\sqrt{3}}\\ 0 & \frac{\sqrt{5}}{4\sqrt{3}} & \frac{5}{18} \end{array}\right]=1-\frac{10}{18}=\frac{4}{9}.$$

The logical entropy is the two-draw probability of drawing a distinction of π, so it could also be computed as the sum of all the distinction probabilities (remembering that a distinction is an ordered pair of elements in different blocks, so the probability of an unordered pair is doubled). Hence, in general, we have: $h\left(\pi \right)=\sum_{\left(u_{i},u_{k}\right)\in \mathrm{dit}\left(\pi \right)}p_{i}p_{k}$, or, in the case at hand:

$$h\left(\pi \right)=2p_{a}p_{b}+2p_{a}p_{c}=2\times \frac{1}{3}\times \frac{1}{4}+2\times \frac{1}{3}\times \frac{5}{12}=\frac{1}{6}+\frac{5}{18}=\frac{4}{9}.$$

There is then a general theorem [2] showing how logical entropy measures measurement.

**Theorem** **2** (set case of measuring measurement)**.** *The increase in logical entropy from h(ρ(π)) to $h(\hat{\rho }\left(\pi \right))$ in the Lüders mixture operation $\hat{\rho }\left(\pi \right)=\sum_{r\in g(U)}P_{g^{-1}\left(r\right)}\rho \left(\pi \right)P_{g^{-1}\left(r\right)}$ is the sum of the squares of the off-diagonal non-zero entries in ρ(π) that were zeroed in the measurement operation $\rho \left(\pi \right)⇝\hat{\rho }\left(\pi \right)$.*

In the example, the logical entropy of the post-measurement state is:

$$h\left(\hat{\rho }\left(\pi \right)\right)=1-\mathrm{tr}\left[\hat{\rho }{\left(\pi \right)}^{2}\right]=1-\mathrm{tr}\left[\begin{array}{ccc} \frac{1}{9} & 0 & 0\\ 0 & \frac{1}{16} & 0\\ 0 & 0 & \frac{25}{144} \end{array}\right]=1-\left(\frac{1}{9}+\frac{1}{16}+\frac{25}{144}\right)=1-\frac{16+9+25}{144}=\frac{94}{144}.$$

The sum of the squares of the non-zero off-diagonal terms (representing the coherences) of ρ(π) that were zeroed (decohered) in $\hat{\rho }\left(\pi \right)$ is:

$$2\times {\left(\frac{\sqrt{5}}{4\sqrt{3}}\right)}^{2}=2\times \frac{5}{48}=\frac{5}{24}$$

and the increase in logical entropy due to the making of distinctions is:

$$h\left(\hat{\rho }\left(\pi \right)\right)-h\left(\rho \left(\pi \right)\right)=\frac{94}{144}-\frac{4}{9}=\frac{94}{144}-\frac{64}{144}=\frac{30}{144}=\frac{5}{24}.✔$$

Furthermore, the quantum case follows *mutatis mutandis*.

**Theorem** **3** (quantum case of measuring measurement)**.** *In the Lüders mixture operation $\hat{\rho }=\sum_{r\in g(U)}P_{V_{r}}\rho P_{V_{r}}$, the increase in quantum logical entropy from h(ρ) to $h(\hat{\rho })$ is the sum of the absolute squares of the off-diagonal entries in ρ that were zeroed in the measurement operation $\rho ⇝\hat{\rho }$.*

The dictionary relating the three basic concepts in the math of QM to their set partitional precursors is given in Table 6.

### 4.5. Other Aspects of QM Mathematics

#### 4.5.1. Commuting, Non-Commuting, and Conjugate Operators

We have seen that a Hermitian operator is the QM math version of a real-valued numerical attribute and that the direct-sum decomposition of the operator’s eigenspaces is the QM math version of the inverse-image partition of the numerical attribute. Let F,G:V→V be two Hermitian operators with the corresponding DSDs of ${\left\{V_{j}\right\}}_{j\in J}$ and ${\left\{W_{j^{\prime }}\right\}}_{j^{\prime }\in J^{\prime }}$. Since the two DSDs are the vector space version of partitions, consider the join-like operation giving the set of non-zero subspaces formed by the intersections $V_{j}\cap W_{j^{\prime }}$. The additional generality gained over the join of set partitions is that these subspaces may not span the whole space *V*. Since the vectors in those intersections are simultaneous eigenvectors of *F* and *G*, let SE be the subspace spanned by the simultaneous eigenvectors of *F* and *G*. The commutator [F,G]=FG−GF:V→V is a linear operator on *V* so it has a kernel ker[F,G] consisting of the vectors *v* such that [F,G]v=0. Then, there is the following theorem:

**Theorem** **4** ([58])**.** *SE=ker[F,G]*

*Since commutativity is defined as ker[F,G]=V, we have the following definitions in terms of the vector space partitions or DSDs:*

- *F and G are commuting if SE=V;*
- *F and G are incompatible if SE≠V;*
- *F and G are conjugate if SE=0 (zero space).*

Since the join-like operation on DSDs yields a set of subspaces that do not necessarily span the whole space, that operation is only the *join* of DSDs in the commuting case—or as Hermann Weyl put it: “Thus, combination [join] of two gratings [DSDs] presupposes commutability …” [59] [p. 257].

The set version of *compatible* partitions for the join operation is simply being defined on the same set. Hence, our thesis gives a complete parallelism between compatible partitions and commuting operators.

**Partition math**: A set of compatible partitions π,σ,…,γ defined by f,g,…,h:U→R is said to be *complete*, i.e., a Complete Set of Compatible Attributes or CSCA, if their join is the partition whose blocks are of cardinality one (i.e., $1_{U}$). Then, the elements u∈U are uniquely characterized by the ordered set of values (f(u),g(u),…,h(u).

**QM math**: A set of commuting observables F,G,…,H is said to be *complete*, i.e., a Complete Set of Commuting Observables or CSCO [60] [p. 57], if the join of their eigenspace DSDs is the DSD whose subspaces are of dimension one. Then, the simultaneous eigenvectors of the operators are uniquely characterized by the ordered set of their eigenvalues.

This is a particularly clear case of showing that the QM math is a word-for-word translation (using the Yoga translation dictionary) of partition math. The partition math may be new to quantum theorists, but the whole translation dictionary (e.g., Table 4, Table 5 and Table 6 and the CSCA to CSCO translation) is not just a set of ‘coincidences’. It shows how the QM math comes from the math of partitions (or equivalence relations), which is the math-to-model definiteness versus indefiniteness or distinguishability versus indistinguishability. That brings us to the Feynman rules.

#### 4.5.2. Feynman’s Treatment of Measurement

The partitional approach to understanding the math of QM shows that the key organizing concepts are indistinctions versus distinctions and its cognates. Those concepts define the notion of logical entropy in the new partition-logical theory of information-as-distinctions ([2,61]). When a particle in a superposition state undergoes an interaction, it is often asked “what characterizes whether it is a measurement or not?” As early as 1951 [62], Richard Feynman gave the analysis of “measurement or not” in terms of distinguishability.

If you could, *in principle*, distinguish the alternative *final* states (even though you do not bother to do so), the total, final probability is obtained by calculating the *probability* for each state (not the amplitude) and then adding them together. If you *cannot* distinguish the final states *even in principle*, then the probability amplitudes must be summed before taking the absolute square to find the actual probability [63] [pp. 3–9].

This analysis has been further explained by John Stachel (see [41] [pp. 110–111] for more analysis).

Feynman’s approach is based on the contrast between processes that are *distinguishable* within a given physical context and those that are *indistinguishable* within that context. A process is distinguishable if some record of whether or not it has been realized results from the process in question; if no record results, the process is indistinguishable from alternative processes leading to the same end result [64] [p. 314].

The same points are restated by Anton Zeilinger using, in effect, the notion of information-as-distinctions. Brukner and Zeilinger also suggest the formula for logical entropy as an information measure [4] [p. 332].

In other words, the superposition of amplitudes … is only valid if there is no way to know, even in principle, which path the particle took. It is important to realize that this does not imply that an observer actually takes note of what happens. It is sufficient to destroy the interference pattern, if the path information is accessible in principle from the experiment or even if it is dispersed in the environment and beyond any technical possibility to be recovered, but in principle still “out there”. The absence of any such information is the essential criterion for quantum interference to appear [65] [p. 484].

What Feynman calls “distinguishability”, Zeilinger calls “information” since, as one of the founders of quantum information theory, Charles Bennett, put it, information “is the notion of distinguishability abstracted away from what we are distinguishing, or from the carrier of information …” [66] [p. 155].

Feynman gives a number of examples ([63] [§ 3-3]; [67] [pp. 17–18]) such as a particle scattering off the atoms in a crystal. If there is no physical record of which atom the particle scattered off of (i.e., the indistinguishable case), then no measurement took place so the *amplitudes* for the superposition state of scattering off the different atoms are added to compute the amplitude of the particle reaching a certain final state. However, if all the atoms had, say, spun up, and scattering off an atom flipped the spin, then a physical record exists (i.e., the distinguishable case), so a measurement took place (or, as Stachel put it: “In my terminology, a registration of the realization of a process must exist for it to be a distinguishable alternative” [64] [p. 314]) and the *probabilities* of scattering off the different atoms are added to compute the probability of reaching a certain final state.

The same analysis applies to the well-known double-slit experiment where the distinguishable case is where there are detectors at the slits (or the slits are distinguished by one being closed) and the indistinguishable case is having no detectors at the open slits. However, the important thing to notice about Feynman’s example is that the measurement is entirely at the quantum level; it involves no macroscopic apparatus. Hence, the Feynman analysis bypasses the whole tortured literature trying to analyze measurement in terms of the “decoherence” induced by a macroscopic measuring devices (e.g., [68]). Of course, the quantum-level physical record in the distinguishable case has to be amplified for humans to record the result, but such macroscopic considerations have no role in quantum *theory*.

The implicit principle in Feynman’s analysis of measurement is:

$$\begin{array}{c} \mathrm{If}\mathrm{the}\mathrm{interaction}\mathrm{distinguishes}\mathrm{between}\mathrm{superposed}\mathrm{eigenstates},\\ \mathrm{then}a\mathrm{state}\mathrm{reduction}\mathrm{is}\mathrm{made}.\\ \mathrm{The}\mathrm{State}\mathrm{Reduction}\mathrm{Principle} \end{array}$$

The mathematics of the State Reduction Principle can be stated in both the set case and the QM case.

**Theorem** **5** (State Reduction Principle—set case)**.** *Measurement is described in the set case by the Lüders mixture operation $\hat{\rho }\left(\pi \right)=\sum_{r\in g(U)}P_{g^{-1}\left(r\right)}\rho \left(\pi \right)P_{g^{-1}\left(r\right)}$. The State Reduction Principle then states that if an off-diagonal entry $\rho {\left(\pi \right)}_{ik}\neq 0$ (i.e., $u_{i}$ and $u_{k}$ are in a same-block superposition), then, if $g\left(u_{i}\right)\neq g\left(u_{k}\right)$ (i.e., the interaction distinguishes $u_{i}$ and $u_{k}$), then $\hat{\rho }{\left(\pi \right)}_{ik}=0$ (i.e., the ‘coherence’ between $u_{i}$ and $u_{k}$ is decohered and a reduction is made).*

**Theorem** **6** (State Reduction Principle—QM case)**.** *Measurement is described in QM by the Lüders mixture operation $\hat{\rho }=\sum_{r\in g(U)}P_{V_{r}}\rho P_{V_{r}}$ (measuring ρ by G). The State Reduction Principle then states that if an off-diagonal entry $\rho_{ik}\neq 0$ (i.e., the G-eigenvectors $|u_{i}\rangle$ and $|u_{k}\rangle$ are in a superposition in ρ), then, if $|u_{i}\rangle$ and $|u_{k}\rangle$ have different G-eigenvalues (i.e., the vectors are distinguished by G), then ${\hat{\rho }}_{ik}=0$ (i.e., the vectors are decohered—this is not the Zeh/Zurek (for all practical purposes) “decoherence” [68], but the old-fashioned change from the coherence of a superposition pure state into a decohered mixture of states—and a reduction is made).*

If no distinctions were made by the interaction, then no ‘measurement’ took place.

#### 4.5.3. Von Neumann’s Type I and Type II Processes

John von Neumann (vN) [69] made their famous distinction between the following processes:

1. Type I process of measurement and state reduction
2. Type II process obeying the Schrödinger equation.

We have seen that the Type I processes of measurement involves distinguishability, i.e., the making of distinctions (like which atom the particle scattered off of), so a natural way to designate the Type II processes would be ones that do *not* make distinctions by preserving distinguishability or indistinguishability. The measure of indistinctness of two quantum states is their overlap or inner product. For instance, íf two states have zero indistinctness (zero inner product), then they are fully distinct (orthogonal). Hence, the natural characterization of a Type II process is one that preserves inner products, i.e., a unitary transformation. The connection to solutions to the Schrödinger equation in Hilbert space math is provided by Stone’s Theorem ([24] [p. 114]; [70]).

The partitional approach highlights the key analytical concepts of indistinctions versus distinctions and the cognate notions of indefiniteness versus definiteness or indistinguishability versus distinguishability. Many people working on quantum foundations seem to ignore those key concepts, and then the division between the measurement and unitary evolution seems unfounded, if not “unbelievable”.

It seems unbelievable that there is a fundamental distinction between “measurement” and “non-measurement” processes. Somehow, the true fundamental theory should treat all processes in a consistent, uniform fashion [71] [p. 245].

**Figure 6:**
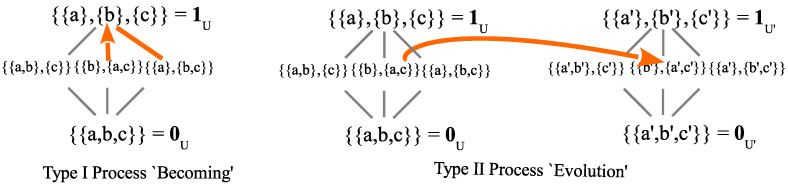
“Anschaulich” (intuitive) picture of two types of quantum processes.

This analysis of a Type I process (indefinite state to more definite state) and a Type II process (change of state at the same level of indefiniteness) in quantum mechanics (see the two arrows in Figure 6) *shows* why there is only one type of fundamental process in classical mechanics.

There are no indefinite states in classical mechanics. Then, it is as if the quantum world below the discrete partition in Figure 6 is not there. Hence, there are no “jumps” (from indefinite to definite states) and there is only the Type II (as it were) process of change, namely from definite states to definite states. Moreover, in the classical no-superposition world, every state is a fully distinguished definite state (a singleton in the classical discrete partition of Figure 6), i.e., an ‘eigenstate’ (as it were), so, an idealized classical measurement is like the quantum measurement of an eigenstate, i.e., it registers the value associated with the state. This is an example of how the *anschaulich* model provided by partition math, e.g., as in Figure 6, reproduces some of the basic and distinctive features of quantum mechanics in the comparison with classical mechanics.

#### 4.5.4. Hermann Weyl’s Imagery for Measurement

An industrial sieve is used to *distinguish* particles of matter of different sizes so it might serve as a helpful metaphor for the quantum process of making distinctions, namely measurement.

In Einstein’s theory of relativity the observer is a man who sets out in quest of truth armed with a measuring-rod. In quantum theory he sets out armed with a sieve [72] [p. 267].

Hermann Weyl quotes Eddington’s passage [59] [p. 255], but uses their own expository notion of a “grating”. Weyl in effect uses the Yoga of Linearization from the mathematical folklore to develop both the set notion of a grating as an “aggregate [which] is used in the sense of ‘set of elements with equivalence relation”’ [59] [p. 239] and the vector space notion of a direct-sum decomposition. In the set-to-vector space move of the Yoga, the “aggregate of *n* states has to be replaced by an *n*-dimensional Euclidean vector space” [59] [p. 256]. The notion of a vector space partition or “grating” in QM is a “splitting of the total vector space into mutually orthogonal subspaces”, so that “each vector $\vec{x}$ splits into *r* component vectors lying in the several subspaces” [59] [p. 256], i.e., a DSD. After thus referring to a partition and a DSD as a “grating” or “sieve”, Weyl notes that “Measurement means application of a sieve or grating” [59] [p. 259], i.e., the making of distinctions by the join-like process described by the Lüders mixture operation.

**Figure 7:**
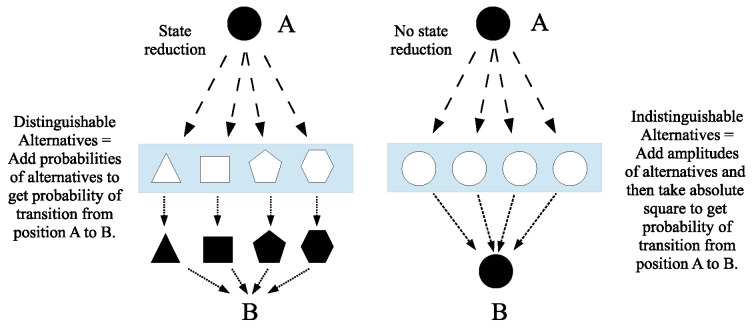
Gratings imagery for Feynman’s rule distinguishable and indistinguishable alternatives.

This imagery of measurement (or non-measurement) as passing through a sieve or grating is illustrated in Figure 7 for Feynman’s rule.

One should imagine the roundish blob of dough as the superposition of the definite shapes in the grating or sieve. The interaction between the superposed blob and the sieve/grating forces a distinction in the state reduction case, so a reduction is made as the blob must pass through one of the definite-shaped holes. In general, a state reduction (‘measurement’) from an indefinite superposition to a more definite state takes place when the particle in the superposition state undergoes an interaction that distinguishes the superposed states (left side of Figure 7). If the grating did not distinguish between the alternatives, then no state reduction takes place in the transition from position *A* to *B* (unitary evolution), as in the Feynman rule for the indistinguishable alternatives “add-amplitudes” case (right side of Figure 7).

### 4.6. A Skeletal Analysis of the Double-Slit Experiment

**Figure 8:**
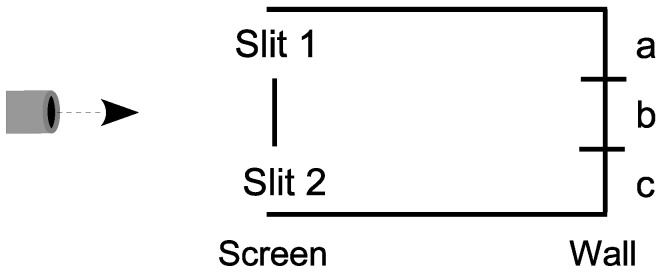
Skeletal setup for the double-slit experiment.

Consider the skeletal case of a particle have three possible states U={a,b,c} which are interpreted as vertical positions in the setup for the double-slit experiment in Figure 8.

In the set-level skeletal analysis, we have discarded the scalars from C, but we are nevertheless left with the scalars 0 and 1, which are the elements of the field $Z_{2}$. There is the natural correspondence between the zero-one vectors in the three-dimensional vector space $Z_{2}^{3}$ (i.e., the column vector ${\left[1,0,0\right]}^{t}$ is associated with {a}, and so forth) which establishes an isomorphism: $Z_{2}^{3}≅℘\left(U\right)$, where the set addition is the symmetric difference, i.e., for S,T∈℘(U), S+T=(S−T)∪(T−S) [73]. That mimics the addition mod 2 in $Z_{2}^{3}$ since, for instance, {a,b}+{b,c}={a,c}. For our dynamics, we assume a non-singular linear transformation $\left\{a\right\}⇝\left\{a^{\prime }\right\}=\left\{a,b\right\}$, $\left\{b\right\}⇝\left\{b^{\prime }\right\}=\left\{a,b,c\right\}$, and $\left\{c\right\}⇝\left\{c^{\prime }\right\}=\left\{b,c\right\}$ which is non-singular, since $\left\{a^{\prime }\right\}=\left\{a,b\right\}$, $\left\{b^{\prime }\right\}=\left\{a,b,c\right\}$, and $\left\{c^{\prime }\right\}=\left\{b,c\right\}$ also form a basis set for ℘(U)—so we also have a partition lattice $\Pi (U^{\prime })$ on the basis set $U^{\prime }=\left\{a^{\prime },b^{\prime },c^{\prime }\right\}$.

The first state reduction takes place as the particle from the left-hand source in Figure 8 arrives at the screen. If the particle does not hit the screen at *b*, then the particle reduces to the superposition |slit1〉+|slit2〉, or in skeletal terms {a,c}.

**Case 1**: There are detectors at the slits to distinguish between the two superposed states, so the state reduces to the half–half mixture of {a} and {c}. The same mixture results if one or the other slit is simply closed. Then, {a} evolves by the non-singular dynamics to {a,b}, which hits the wall and reduces to {a} or {b} with half–half probability. Similarly, {c} evolves to {b,c}, which hits the wall and reduces to {b} or {c} with half–half probability. Since this is the case of distinctions between the alternative paths to {a}, {b}, or {c}, we add the probabilities to obtain:

$$\begin{array}{c} \mathrm{Pr}(\{a\}\;\mathrm{at}\;\mathrm{wall}|\{a,c\}\;\mathrm{at}\;\mathrm{screen})\\ =\mathrm{Pr}\left(\left\{a\right\}\;\mathrm{at}\;\mathrm{wall}|\left\{a\right\}\;\mathrm{at}\;\mathrm{screen}\right)\mathrm{Pr}\left(\left\{a\right\}\;\mathrm{at}\;\mathrm{screen}|\left\{a,c\right\}\;\mathrm{at}\;\mathrm{screen}\right)=\frac{1}{2}\times \frac{1}{2}=\frac{1}{4}.\\ \mathrm{Pr}(\{b\}\;\mathrm{at}\;\mathrm{wall}|\{a,c\}\;\mathrm{at}\;\mathrm{screen})\\ =\mathrm{Pr}(\{b\}\;\mathrm{at}\;\mathrm{wall}|\{a\}\;\mathrm{at}\;\mathrm{screen})\mathrm{Pr}(\{a\}\;\mathrm{at}\;\mathrm{screen}|\{a,c\}\;\mathrm{at}\;\mathrm{screen})\\ +\mathrm{Pr}\left(\left\{b\right\}\;\mathrm{at}\;\mathrm{wall}|\left\{c\right\}\;\mathrm{at}\;\mathrm{screen}\right)\mathrm{Pr}\left(\left\{c\right\}\;\mathrm{at}\;\mathrm{screen}|\left\{a,c\right\}\;\mathrm{at}\;\mathrm{screen}\right)=\frac{1}{2}\times \frac{1}{2}+\frac{1}{2}\times \frac{1}{2}=\frac{1}{2}.\\ \mathrm{Pr}(\{c\}\;\mathrm{at}\;\mathrm{wall}|\{a,c\}\;\mathrm{at}\;\mathrm{screen})\\ =\mathrm{Pr}\left(\left\{c\right\}\;\mathrm{at}\;\mathrm{wall}|\left\{c\right\}\;\mathrm{at}\;\mathrm{screen}\right)\mathrm{Pr}\left(\left\{c\right\}\;\mathrm{at}\;\mathrm{screen}|\left\{a,c\right\}\;\mathrm{at}\;\mathrm{screen}\right)=\frac{1}{2}\times \frac{1}{2}=\frac{1}{4}. \end{array}$$

**Figure 9:**
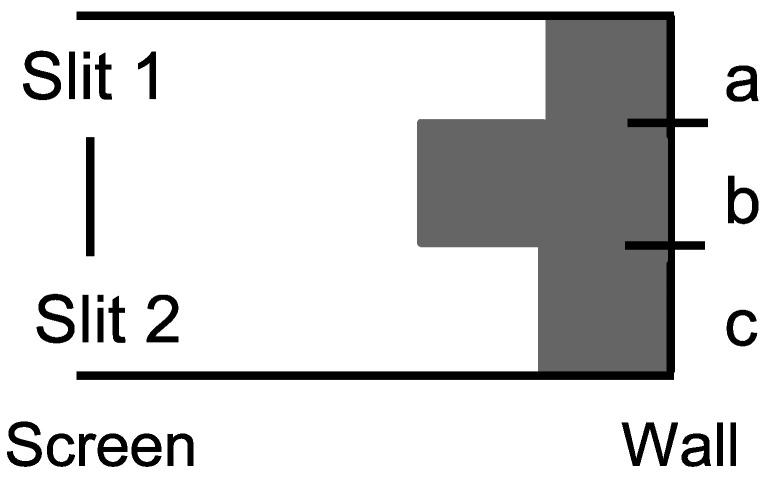
Probabilities at the wall with distinctions at the screen.

Hence, the probability distribution in the Case 1 of measurement or distinguishing at the screen is given in Figure 9.

**Case 2**: There are no detectors to distinguish between the (open) slits in the superposition {a,c}, so it linearly evolves by the dynamics {a,c}={a}+{c}⇝{a,b}+{b,c}={a,c}. Hence, the probabilities at the wall are:

$$\begin{array}{c} \mathrm{Pr}(\{a\}\;\mathrm{at}\;\mathrm{wall}\;|\{a,c\}\;\mathrm{at}\;\mathrm{screen})\\ =\mathrm{Pr}\left(\left\{a\right\}\;\mathrm{at}\;\mathrm{wall}|\left\{a,c\right\}\;\mathrm{at}\;\mathrm{wall}\right)\mathrm{Pr}\left(\left\{a,c\right\}\;\mathrm{at}\;\mathrm{wall}|\left\{a,c\right\}\;\mathrm{at}\;\mathrm{screen}\right)=\frac{1}{2}\times 1=\frac{1}{2}.\\ \mathrm{Pr}(\{b\}\;\mathrm{at}\;\mathrm{wall}|\{a,c\}\;\mathrm{at}\;\mathrm{screen})\\ =\mathrm{Pr}(\{b\}\;\mathrm{at}\;\mathrm{wall}|\{a,c\}\;\mathrm{at}\;\mathrm{wall})\mathrm{Pr}(\{a,c\}\;\mathrm{at}\;\mathrm{wall}|\{a,c\}\;\mathrm{at}\;\mathrm{screen})=0\times 1=0.\\ \mathrm{Pr}(\{c\}\;\mathrm{at}\;\mathrm{wall}\;|\{a,c\}\;\mathrm{at}\;\mathrm{screen})\\ =\mathrm{Pr}\left(\left\{c\right\}\;\mathrm{at}\;\mathrm{wall}|\left\{a,c\right\}\;\mathrm{at}\;\mathrm{wall}\right)\mathrm{Pr}\left(\left\{a,c\right\}\;\mathrm{at}\;\mathrm{wall}|\left\{a,c\right\}\;\mathrm{at}\;\mathrm{screen}\right)=\frac{1}{2}\times 1=\frac{1}{2}. \end{array}$$

**Figure 10:**
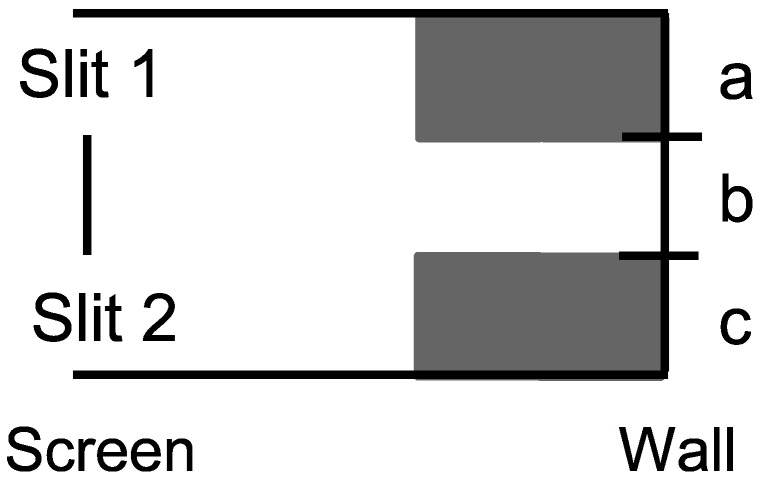
Probabilities at the wall with no distinctions at the screen.

Hence, the probability distribution in the Case 2 of no distinctions at the screen is given in Figure 10.

The Case 2 distribution shows the usual probability stripes due to the interference in the linear evolution of the superposition state {a,c}, i.e., the destructive interference in the evolved superposition {a,b}+{b,c}={a,c}.

**Figure 11:**
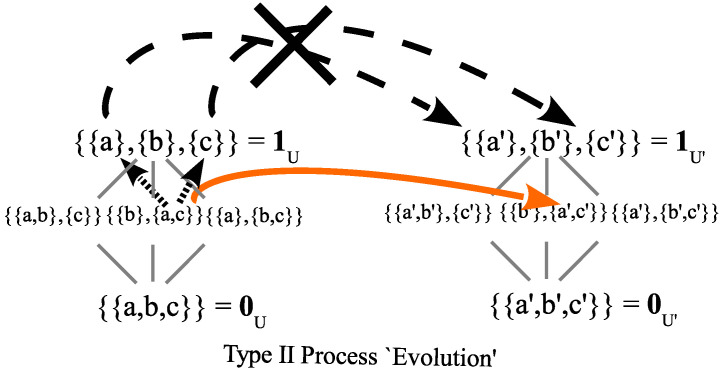
Case 2 evolution taking place at a non-classical level of indefiniteness.

Our classical intuitions insist on asking “Which slit did the particle go through in Case 2?”. That question assumes that the evolution of the state {a,c} was at the classical level where the slits were distinguished. However, in Case 2, the slits were not distinguished so the evolution took place at the lower level in the skeletal lattice of partitions. In Figure 11, the Case 2 (and vN Type II) evolution (solid arrow) is illustrated as going from the superposition state {a,c} in the partition lattice of states on U={a,b,c}, to the superposition state $\left\{a^{\prime },c^{\prime }\right\}$ lattice of states on $U^{\prime }=\left\{a^{\prime },b^{\prime },c^{\prime }\right\}$. The Case 1 (and vN Type I) becoming to one of the classical states {a} or {c}, followed by the evolution of definite states (dashed arrows X-ed out) does not take place with no detection at the slits.

The important ‘take-away’ is that there are different levels of indefiniteness (as illustrated in the partition lattice) and evolution can take place at a non-classical level of indefiniteness, so, in that case, there is *no matter of fact* of the particle going through one slit {a} or the other {c} *at the classical level*.

**Figure 12:**
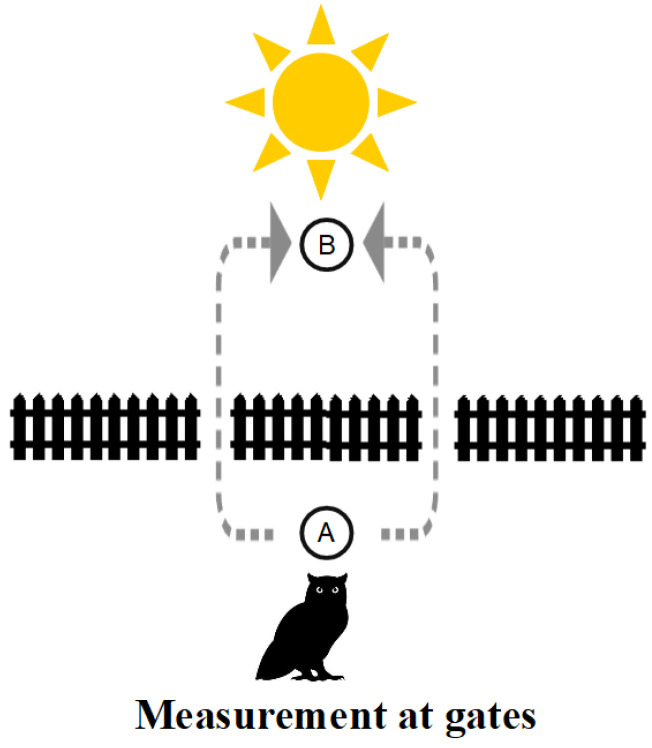
Measurement at gates (owl has to walk from *A* to *B* through one of the gates).

Sometimes, metaphors can serve as an aid or crutch to our biologically evolved intuitions. Suppose we have a fence across a field with two gates (like in the double slit experiment). When the sun is shining, that is a metaphor for detecting which gate a creature may walk through to get from *A* to *B*. Our creature is Hegel’s Owl of Minerva who only flies at night [74] [p. 13]. Hence, when the sun shines, the owl is limited to “flatland” [75], so it has to go through one gate or another to get from *A* to *B* as in Figure 12.

**Figure 13:**
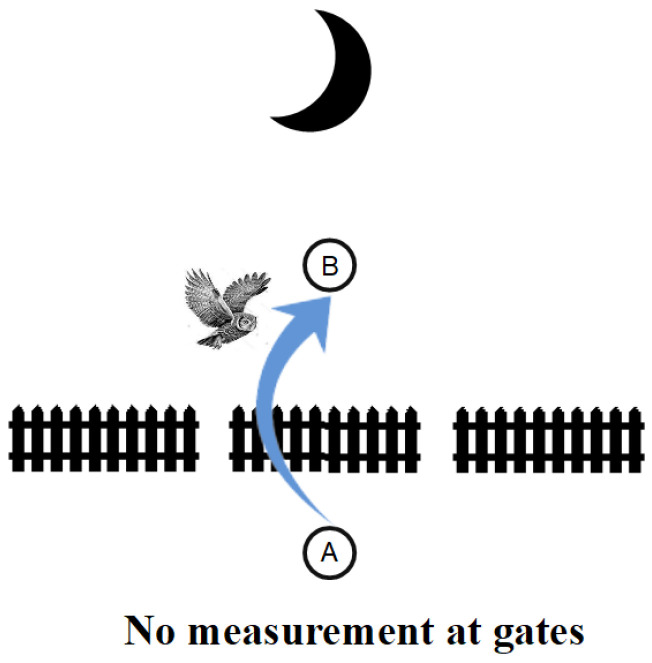
No measurement at gates (owl can fly from *A* to *B* without going through a gate).

However, when the sun sets, the Owl of Minerva’s “flights and perches” [76] [p. 198] can go from ground perch *A* to ground perch *B* without going through one gate or the other, as in Figure 13.

Our classical (“flatlander”) intuitions see only the definite ground-level paths or trajectories and, in the absence of the projecting light source (or sun) as in Case 2 above, will insist on asking “Which gate did the owl go through?”. However, with no detections at the slits in the double-slit experiment, there is *no matter of fact* of the particle going through a slit at the classical level, since the evolution is at the non-classical quantum level, as in Figure 11 (illustrated by the third dimension in our flatlander metaphor of Figure 13).

Quantum states are built from below, as it were (remember the 3D printing metaphor), through being in-formed by information-as-distinctions. Von Neumann’s two processes separate the Type I *becoming* from below between levels of indefiniteness and the Type II unitary *evolution* at the same level of indefiniteness. Figure 11 shows how quantum evolution transforms one superposition into another at the same level of indefiniteness (which allows interference) *without* any becoming or rising to the level of classical fully distinguished states. That is hard to understand for those who insist on “interpreting” QM with *only* our evolved intuitions based on the macroscopic (superposition-less) world.

## 5. Discussion

Our thesis is that the math of QM is the Hilbert space version of the math of partitions, or, put the other way around, the math of partitions is the skeletonized version of QM math. There are many other aspects of QM math that could be investigated, such as group representations on sets or on vector spaces over C, since a group is essentially a ‘dynamic’ algebraic way to define an equivalence relation or DSD ([77] and [59] [p. 73]) (e.g., the orbit partition in a set representation or the DSD of irreducible subspaces in the vector space over C representation). However, in this introductory treatment, we have hopefully analyzed enough aspects of QM math to illustrate our thesis.

The main mystery in QM is superposition (entanglement being a particularly mysterious special case) and the quantum state of becoming, wherein a superposition state jumps to a more definite state by being in-formed by distinctions. The rules, governing when an interaction involving a superposition constitutes a state reduction or not, are given by the Feynman rules based on the notion of distinguishability or indistinguishability. At the logical level, the mathematics of distinguishability versus indistinguishability (or distinctions versus indistinctions) is the math of partitions. The quantitative version of partitions gives the notion of logical entropy that is the measure of information-as-distinctions, so that, in the Type I measurement process, distinctions in-form an indefinite state to jump to a more definite state. This treatment of measurement as “more-definite its from dits” is perhaps the best way to understand Wheeler’s “its from bits” mantra [78]. Furthermore, if information has that role, then Rota’s idea, that is, information is to partitions as probability is to subsets, connects partitions to understanding QM. In contrast to the usual imagery of superposition as the addition of waves, the notion of ontological indefiniteness provides the alternative characteristic feature of non-classical superposition states. The classroom ripple-tank model of the double-slit experiment is doubly misleading in modeling the wave function as a matter (water) wave (instead of a non-ontic computational device or “probability wave”) and in treating the no-detection evolving-superposition Case 2 as “going through both slits at once”.

However, it will be said, “from the logical viewpoint, a property or the complementary property has to definitely apply to a particle”. That is the viewpoint of the classical logic of subsets; an element has to be in a subset or in its complement; there is no indefiniteness. There is, however, an equally fundamental logic at the mathematical level, the dual logic of partitions (See Table 1). In a (non-discrete) partition on the definite- or eigenstates of an observable, each non-singleton block represents the (skeletonized version) of objective indefiniteness of those eigenstates in the block. In a state reduction, distinctions are made to create more definiteness—with the classical mixed state of fully distinguished states being the maximal result. That is the notion of becoming described in partition logic, not the sort of definite-to-definite transformations considered in classical physics.

Subsets and partitions are equally fundamental (indeed, dual) logical concepts. The notion of partitions (or equivalence relations) is the logical concept to describe indistinctions versus distinctions, indistinguishability versus distinguishability, or indefiniteness versus definiteness. Thus, the partitional approach to QM does not use some jury-rigged concepts (e.g., Bohmian mechanics or spontaneous localization theory) to have yet another “interpretation” of QM. It should not be surprising that fundamental logical concepts can explicate the fundamental physical theory [79].

This approach to better understanding or interpreting QM works with the standard von Neumann/Dirac quantum theory. It does not involve any new physics. In that sense, the partitional approach shows how to develop Shimony’s idea of the *Literal Interpretation* of the math or “formalism of quantum mechanics” [8] [pp. 6–7]. Furthermore, the partitional analysis substantiates the analysis of Heisenberg, Shimony, Jaeger, and others which describes the quantum world in terms of potentialities or latencies, where, in both cases, the key attribute was the *reality* of objective indefiniteness.

The conceptual elements of quantum theory that now underlie our picture of the physical world include objective chance, quantum interference, and the objective indefiniteness of dynamical quantities. Quantum interference, which is directly observable, was readily absorbed by the physics community. Objective chance and indefiniteness, being of more philosophical significance, gained acceptance only after much debate and conceptual analysis, when it was recognized that observed phenomena are better understood through these notions than through older ones or hidden variables [41] [p. vii].

A non-philosophical quantum physicist might consider the following simple litmus test. “Does a superposition state (in the measurement basis) objectively have a definite or indefinite value before measurement?”. If the answer is “objectively indefinite”, then the physicist is essentially using the vision of quantum reality analyzed here.

The findings of this paper can be summarized as follows:

- Superposition is the key non-classical notion of state in QM math;
- Objective indefiniteness is its ontological counterpart;
- The quantum notion of becoming (vN Type I) is the jump from an indefinite state to a more definite state;
- The quantum notion of evolution (vN Type II) is between states at the same level of indefiniteness.

This way of understanding or interpreting quantum mechanics might be called the *Objective Indefiniteness Interpretation* [58].

## Figures and Tables

**Table 1:** Elements and distinctions (its and dits) duality between the two algebras.

Algebra of subsets ℘(U) of *U*	Algebra of partitions Π(U) on *U*
Its = elements of subsets	Dits = distinctions of partitions
P. O. inclusion of subsets S⊆T	P. O. inclusion of ditsets dit(σ)⊆dit(π)
Join: S∨T=S∪T	Join: dit(σ∨π)=dit(σ)∪dit(π)
Top: subset *U* with all elements	Top: partition $1_{U}$ with all possible dits
Bottom: subset ∅ with no elements	Bottom: partition $0_{U}$ with no dits

**Table 2:** Set and quantum versions of non-determinate and determinate results.

Choice Function	Quantum Measurement (Maximal)
f({a,b,…,c})=b	$\alpha |a\rangle +\beta |b\rangle +\cdots +\gamma |c\rangle \overset{\mathrm{indeterminate}}{⇝}|b\rangle$
f({b})=b	$|b\rangle \overset{\mathrm{determinate}}{⇝}|b\rangle$

**Table 3:** Corresponding partition and quantum concepts.

Partition Concept	Corresponding Quantum Concept
Non-singleton block, e.g., {a,b}	Superposition pure state
Indiscrete partition $0_{U}=\left\{\left\{a,b,c,d\right\}\right\}$	Largest pure state
Singleton block, e.g., {d}	Classical state (no superposition)
Discrete partition $1_{U}=\left\{\left\{a\right\},\left\{b\right\},\left\{c\right\},\left\{d\right\}\right\}$	Mixture of classical states

**Table 4:** Quantum state ρ as Hilbert space version of partition.

Partition Math	Hilbert Space Math
Density matrix: ρ(π)	ρ
Orthonormal vectors: $\langle b_{j^{\prime }}|b_{j}\rangle =\delta_{jj^{\prime }}$	$\langle u_{i^{\prime }}|u_{i}\rangle =\delta_{ii^{\prime }}$
Eigenvalues: $\mathrm{Pr}\left(B_{1}\right),\ldots ,\mathrm{Pr}\left(B_{m}\right),0,\ldots ,0$	$\lambda_{1},\ldots ,\lambda_{n}$
Spectral decomposition: $\rho \left(\pi \right)=\sum_{j=1}^{m}\mathrm{Pr}\left(B_{j}\right)|b_{j}\rangle \langle b_{j}|$	$\rho =\sum_{i=1}^{n}\lambda_{i}|u_{i}\rangle \langle u_{i}|$
Non-zero off-diagonals: indistinction in a block $B_{j}$	Coherence in a superposition

**Table 5:** Set-level concepts and the corresponding vector (Hilbert) space concepts.

Set Concept	Vector-Space Concept
Partition ${\left\{f^{-1}\left(r\right)\right\}}_{r\in f(U)}$	DSD ${\left\{V_{r}\right\}}_{r\in f(U)}$
$U={⊎}_{r\in f(U)}f^{-1}\left(r\right)$	$V={⊕}_{r\in f(U)}V_{r}$
Numerical attribute f:U→R	Observable $Fu_{i}=f\left(u_{i}\right)u_{i}$
f↾S=rS	$Fu_{i}=ru_{i}$
Constant set *S* of *f*	Eigenvector $u_{i}$ of *F*
Value *r* on constant set *S*	Eigenvalue *r* of eigenvector $u_{i}$
Characteristic fcn. $\chi_{S}:U\to \left\{0,1\right\}$	Projection operator $P_{\left[S\right]}u_{i}=\chi_{S}\left(u_{i}\right)u_{i}$
$\cup_{r\in f(U)}\left(f^{-1}\left(r\right)\cap \left(\right)\right)=I:℘\left(U\right)\to ℘\left(U\right)$	$\sum_{r\in f(U)}P_{V_{r}}=I:V\to V$
Spectral Decomp. $f=\sum_{r\in f(U)}r\chi_{f^{-1}\left(r\right)}$	Spectral Decomp. $F=\sum_{r\in f(U)}rP_{V_{r}}$
Set of *r*-constant sets $℘(f^{-1}\left(r\right))$	Eigenspace $V_{r}$ of *r*-eigenvectors
Cartesian product $U\times U^{\prime }$	Tensor product $V⊗V^{\prime }$

**Table 6:** Three basic notions: set version and corresponding Hilbert space version.

Partition Math	Hilbert Space Math
State: $\rho \left(\pi \right)=\sum_{j=1}^{m}\mathrm{Pr}\left(B_{j}\right)|b_{j}\rangle \langle b_{j}|$	$\rho =\sum_{i=1}^{n}\lambda_{i}|u_{i}\rangle \langle u_{i}|$
Observable: $g=\sum_{r\in g(U)}r\chi_{g^{-1}\left(r\right)}:U\to R$	$G=\sum_{r\in g(U)}rP_{V_{r}}$
Measurement: $\hat{\rho }\left(\pi \right)=\sum_{r\in g(U)}P_{g^{-1}\left(r\right)}\rho \left(\pi \right)P_{g^{-1}\left(r\right)}$	$\hat{\rho }=\sum_{r\in g(U)}P_{V_{r}}\rho P_{V_{r}}$

## Data Availability

No new data were created or analyzed in this study. Data sharing is not applicable to this article.

## Abbreviations

The following abbreviations are used in this manuscript:

CSCA	Complete Set of Compatible Attributes
CSCO	Complete Set of Commuting Observables
PII	Principle of Identity of Indistinguishables
QM	Quantum Mechanics
